# Unravelling the Function of the Sesquiterpene Cyclase STC3 in the Lifecycle of *Botrytis cinerea*

**DOI:** 10.3390/ijms25105125

**Published:** 2024-05-08

**Authors:** Víctor Coca-Ruiz, Ivonne Suárez, Josefina Aleu, Jesús M. Cantoral, Celedonio González, Carlos Garrido, Nélida Brito, Isidro G. Collado

**Affiliations:** 1 Departamento de Química Orgánica, Facultad de Ciencias, Universidad de Cádiz, 11510 Puerto Real, Cádiz, Spain; victor.coca@uca.es (V.C.-R.); ivonne.suarez@uca.es (I.S.); josefina.aleu@uca.es (J.A.); 2 Instituto de Investigación en Biomoléculas (INBIO), Universidad de Cádiz, 11510 Puerto Real, Cádiz, Spain; 3 Laboratorio de Microbiología, Departamento de Biomedicina, Biotecnología y Salud Pública, Facultad de Ciencias del Mar y Ambientales, Universidad de Cádiz, 11510 Puerto Real, Cádiz, Spain; jesusmanuel.cantoral@uca.es (J.M.C.); carlos.garrido@uca.es (C.G.); 4 Instituto de Investigación Vitivinícola y Agroalimentaria (IVAGRO), Universidad de Cádiz, 11510 Puerto Real, Cádiz, Spain; 5 Área de Bioquímica y Biología Molecular, Departamento de Bioquímica, Microbiología, Biología Celular y Genética, Universidad de La Laguna, 38200 San Cristóbal de La Laguna, Tenerife, Spain; cglez@ull.edu.es

**Keywords:** *Botrytis cinerea*, sesquiterpene cyclase, secondary metabolism, metabolites and sesquiterpenes

## Abstract

The genome sequencing of *Botrytis cinerea* supplies a general overview of the map of genes involved in secondary metabolite synthesis. *B. cinerea* genomic data reveals that this phytopathogenic fungus has seven sesquiterpene cyclase (*Bcstc*) genes that encode proteins involved in the farnesyl diphosphate cyclization. Three sesquiterpene cyclases (BcStc1, BcStc5 and BcStc7) are characterized, related to the biosynthesis of botrydial, abscisic acid and (+)-4-epi-eremophilenol, respectively. However, the role of the other four sesquiterpene cyclases (BcStc2, BcStc3, BcStc4 and BcStc6) remains unknown. BcStc3 is a well-conserved protein with homologues in many fungal species, and here, we undertake its functional characterization in the lifecycle of the fungus. A null mutant Δ*Bcstc3* and an overexpressed–*Bcstc3* transformant (Ov*Bcstc3*) are generated, and both strains show the deregulation of those other sesquiterpene cyclase-encoding genes (*Bcstc1*, *Bcstc5* and *Bcstc7*). These results suggest a co-regulation of the expression of the sesquiterpene cyclase gene family in *B. cinerea*. The phenotypic characterization of both transformants reveals that BcStc3 is involved in oxidative stress tolerance, the production of reactive oxygen species and virulence. The metabolomic analysis allows the isolation of characteristic polyketides and eremophilenols from the secondary metabolism of *B. cinerea*, although no sesquiterpenes different from those already described are identified.

## 1. Introduction

Sesquiterpenes are C15 terpenes consisting of three isoprene units, found in linear (as farnesene), monocyclic (as zingiberene and humulene), bicyclic (as caryophyllene), tricyclic (as longifolene) and tetracyclic (as punctaporonin C) forms. They are produced by higher plants, marine organisms and fungi [1,2]. The biological activity of many of these natural products is related to human health. For instance, arvestolides H and I and artefreynic acids B, C, and G inhibit the production of nitric oxide [3,4]; chrysanthemulide A has anti-inflammatory activity [5]; 14-*O*-acetylinsulicolide A, 6β,9α-dihydroxy-14-*p*-nitrobenzoylcinnamolide and insulicolide A are cytotoxic against renal carcinoma cell lines [6]; and santhemoidin A is active against *Trypanosoma brucei* rhodesiense trypomastigotes [7]. Moreover, sesquiterpenes have been recognized as replacements for petroleum-derived jet engine fuels [8]. These findings highlight the need to deepen the analysis of sesquiterpene biosynthesis due to their commercial interest.

The sesquiterpene synthases (EC:4.2.3.-) are a group of enzymes that convert the linear C15 precursor farnesyl diphosphate (FDP) into a diverse class of natural products. Many sesquiterpenes are cyclic compounds, and the first reaction in their biosynthesis is the cyclization of FDP at the proximal C6-C7 or the distal C10-C11 double bond. Therefore, the synthases involved in this step are also named sesquiterpene cyclases (STC) [9,10,11,12]. Subsequent modifications such as oxidations or substitution with different functional groups, a lactone ring and rearrangements of the 15-carbon skeleton produce the related sesquiterpenoids [13,14].

In fungi, these compounds constitute the most abundant and diverse group of all categories in the vast system of natural products [15]. Many act as phytotoxins in the interaction between phytopathogenic fungi and their host [16,17]. Botrydial is one example of these non-host-specific toxic sesquiterpenes. It is produced by the phytopathogenic fungus *B. cinerea*, the causal agent of gray mold, classified as the second most relevant plant pathogen for its economic and scientific importance [18]. Botrydial is secreted during the fungus–host interaction, causing chlorosis and cell collapse, and its role in virulence is closely related to the polyketide botcinin, the second major phytotoxin of *Botrytis* [19,20,21]. It triggers the hypersensitive response of plants to pathogens, modulated by salicylic acid and jasmonic acid signaling, that produces a rapid localized cell death [22]. Botrydial also induces phosphatidic acid production, a phospholipid second messenger involved in the induction of plant defense responses [23]. Moreover, this phytotoxin also modulates the interaction of *B. cinerea* with other fungi and bacteria and their environment [24,25].

Over the last several years, our group has been studying the sesquiterpene synthesis by *B. cinerea* [21]. We found six *Bcstc* genes in the *B. cinerea* B05.10 strain (*Bcstc1*/*Bcbot2*, *Bcstc2*, *Bcstc3*, *Bcstc4*, *Bcstc5* or *Bcaba5* and *Bcstc7*) that encode proteins with the two conserved magnesium-binding sites required for FDP cyclization. A seventh *Bcstc* gene, *Bcstc6* (Gene ID BofuT4P10000109001), is specific to the T4 strain and absent in B05.10 [26]. We functionally characterized the sesquiterpene cyclases BcStc1, BcStc5 and BcStc7. *Bcstc1*/*Bcbot2* encodes for a pentalenene cyclase, which catalyzes the first step in the biosynthesis of the botrydial phytotoxin and other important derived sesquiterpenes, generating the precursor tricyclic alcohol presilphiperfolan-8β-ol [27,28]. The deletion of the *Bcsct5* gene in the ABA-overproducer strain ATCC58025 let us conclude that BcStc5/BcAba5 is the key enzyme responsible for the sesquiterpenoid abscisic acid biosynthesis [29], and BcStc7 is involved in (+)-4-epi-eremophilenol biosynthesis, a member of the family of eremophilene sesquiterpenes [30,31,32]. Finally, the *Bcstc2* gene is related to the cryptic metabolite botrycinereic acid synthesis, although the role of BcStc2 is not fully understood yet [33]. The role of the other sesquiterpene cyclases of *B. cinerea* is still unknown. To date, BcStc2, BcStc3 and BcStc4 remain uncharacterized.

Here, we achieve the functional characterization of the sesquiterpene cyclase 3 (BcStc3). We describe the *Bcstc3* expression pattern in vitro and in planta and investigate the putative role of the protein in the virulence and biology of *B. cinerea*, generating a *Bcstc3* knock-out mutant and a new strain that constitutively expresses the *Bcstc3* gene.

## 2. Results

### 2.1. BcStc3 Is a Well-Conserved Protein

BcStc3 has 411 amino acid residues with a theoretical molecular weight of 486,551.89 g/mol and an isoelectric point of 6.34, estimated using the Expasy Protparam tool (https://web.expasy.org/protparam/ (accessed on 1 December 2023)). Its aliphatic index is predicted to be 90.73, and the grand average of hydropathicity (GRAVY) is −0.381, indicating that BcStc3 is a thermostable soluble protein located in the cell cytoplasm, according to DeepLoc (version v2.0) (https://services.healthtech.dtu.dk/services/DeepLoc-2.0/ (accessed on 1 December 2023)) prediction. A terpene synthase family 2, C-terminal metal-binding domain (Pfam 19086, at 170–336 amino acid residues) was identified using the InterProScan server (https://www.ebi.ac.uk/interpro/ (accessed on 30 January 2024)) [34] and as a terpene cyclase nonplant C1 protein (cd00687, from 147 to 336 residue) according to the Conserved Domain Database (CDD) from NCBI [35,36]. Both domains were also found in the BcStc1, BcSct4 and BcStc5 proteins (Table 1), but BcStc3 showed the highest sequence identity percentage with BcStc4 (23.84%). The other two members of the BcStc protein family of *B. cinerea* B05.10 (BcStc2 and BcStc7) were grouped into the trichodiene synthase or TRI5 family (Pfam 06330 domain), and the percentage identity values with the other four family proteins ranged between 11.21 and 20.66% (Table 1).

**Figure 1:**
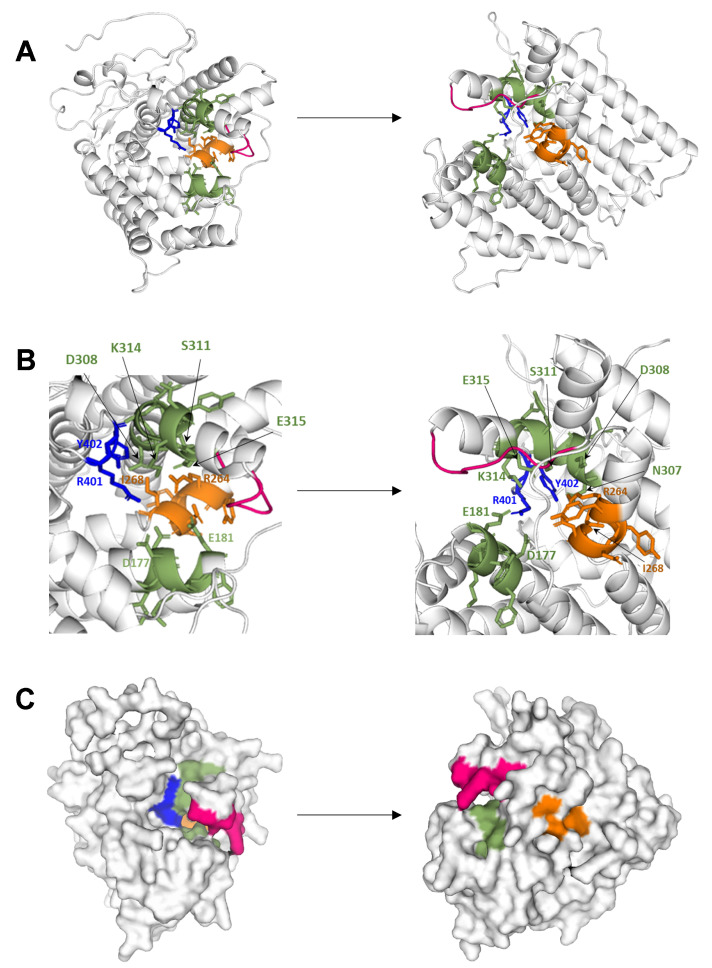
Tertiary structure of the protein BcStc3 via AlphaFold modeling. (**A**) Cartoon representations highlighting the two magnesium binding motifs (green), the effector triad (orange) and the H1α-loop (red). The R401 and Y402 residues, involved in substrate binding, are shown as sticks (blue). (**B**) Close-up view of the active site of BcStc3. The canonical conserved residues are shown as sticks. (**C**) Surface views of BcStc3; the conserved motifs are colored as in (**A**).

The conservation profile of BcStc3 protein amino acid residues showed a low conservation of residues in the N-terminal region, but different canonical motifs of the class I terpenoid cyclases [37,38] were identified at the C-terminus region (Supplementary Figure S1). Two conserved motifs involved in the binding of the magnesium cations were found: an aspartic-rich motif, DDMWE, at amino acid 177, and an NSE/DTE motif, NDLASYDKE, at amino acid 307 (Supplementary Figure S1). Moreover, the conserved effector triad was identified at R264-D267-I268 (Supplementary Figure S1), and the RY conserved pair related to the binding of farnesyl pyrophosphate [39,40] was located at the C-terminus of the protein (residues 401 and 402, respectively) (Figure 1). The molecular model of the BcStc3 protein was generated using the Alphafold tool (https://alphafold.ebi.ac.uk/ (accessed on 8 December 2023)) (version v2.1.2) [41], finding the α-helical fold (Figure 1) typical for class I terpene synthases [37,38]. As expected, the arrangement of the conserved motifs identified in the sequence defines the catalytic site of the protein (Figure 1). Furthermore, the H-α1 loop, a conserved structural motif that shields the active site and stabilizes the closed enzyme conformation after the substrate binding [42], and the conserved asparagine residue (N329) at the C-terminus of the H-1α loop were also identified in the BcStc3 model (Figure 1).

**Table 1:** BcStc protein family of *B. cinerea* B05.10.

Gene ID ^a^	Accession Number ^b^	Protein Name	% Identity ^c^						aa	pfam ^d^	Name ^e^	EC Number ^e^	Reaction (IUBMB) ^e^
Bcin12g06390	XP_024552383.1	BcStc1/BcBot2	100.00						399	PF19086	presilphiperfolanol synthase	4.2.3.74	(2E,6E)-farnesyl diphosphate + H_2_O = presilphiperfolan-8β-ol + diphosphate
Bcin08g02350	XP_001551948.1	BcStc2	13.06	100.00					329	PF06330	trichodiene synthase	4.2.3.6	(2E,6E)-farnesyl diphosphate = trichodiene + diphosphate
Bcin13g05830	XP_024552712.1	BcStc3	14.28	15.80	100.00				411	PF19086	aristolochene synthase	4.2.3.9	(2E,6E)-farnesyl diphosphate = aristolochene + diphosphate
Bcin04g03550	XP_001546971.2	BcStc4	16.54	20.66	23.84	100.00			441	PF19086	ophiobolin F synthase	4.2.3.145	(2E,6E,10E,14E)-geranylfarnesyl diphosphate + H_2_O = ophiobolin F + diphosphate
Bcin01g03520	XP_001550978.1	BcStc5	19.19	11.76	13.93	16.09	100.00		323	PF19086	fusicocca-2,10(14)-diene synthase	4.2.3.43	geranylgeranyl diphosphate = fusicocca-2,10(14)-diene +diphosphate
Bcin11g06510	XP_024551950.1	BcStc7	12.77	17.44	14.95	14.33	11.21	100.00	321	PF06330	trichodiene synthase	4.2.3.6	(2E,6E)-farnesyl diphosphate = trichodiene + diphosphate
			BcStc1/ BcBot2	BcStc2	BcStc3	BcStc4	BcStc5	BcStc7					

^a^ Gene ID from Ensembl Fungi database (http://fungi.ensembl.org/ (accessed on 22 January 2024)). ^b^ Protein ID from on NCBI database (http://www.ncbi.nlm.nih.gov (accessed on 22 January 2024)). ^c^ Sequence identities were obtained using Sequence Identity And Similarity online tool (SIAS—http://imed.med.ucm.es/Tools/sias.html (accessed on 22 January 2024)) from the inmunomedicine group of the Complutense University of Madrid, Madrid, Spain). ^d^ Pfam domain signatures were found using the InterProScan server (https://www.ebi.ac.uk/interpro/ (accessed on 30 January 2024)) [34] and Conserved Domain Database (CDD) from NCBI [43]. ^e^ Protein sequences were annotated using KEGG BlastKoala annotation tool (KEGG Orthology and Links Annotation—https://www.kegg.jp/blastkoala/ (accessed on 23 January 2024)) [44].

**Figure 2:**
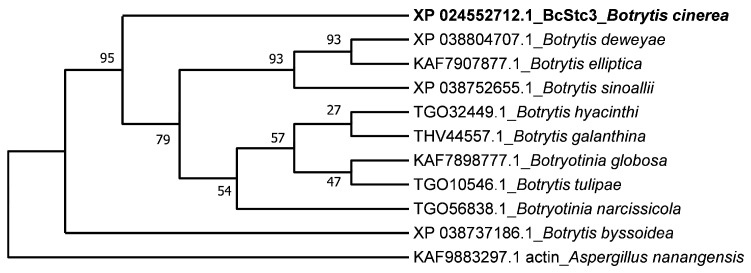
Phylogenetic tree of BcStc3 and homologous protein sequences of other *Botrytis* species. The phylogenetic tree was inferred using the neighbor-joining method via MEGA11 software (version v11.0) with bootstrap values for 1000 replicates which are indicated. Protein sequences were selected after running similarity search by BlastP (https://blast.ncbi.nlm.nih.gov/Blast.cgi?PAGE=Proteins (accessed on 26 January 2024)) using BcStc3 as query sequence. NCBI accession number of each sequence is shown. Actin (KAF9883297.1) of *A. nanangensis* was used as outgroup.

The putative conservation of BcStc3 in *Botrytis* species was analyzed. A BlastP (https://blast.ncbi.nlm.nih.gov/Blast.cgi?PAGE=Proteins (accessed on 26 January 2024)) search against the NCBI non-redundant protein sequences database restricted to the genera *Botrytis* (taxid: 33196) and *Botryotinia* (taxid: 40558) was performed using BcStc3 as a query sequence. Homologous proteins were found in *Botrytis tulipae*, *Botrytis sinoallii*, *Botrytis elliptica*, *Botrytis deweyae*, *Botrytis galanthina*, *Botrytis hyacinthi*, *Botrytis byssoidea*, *Botryotinia globosa* and *Botryotinia narcissicola*, with a percent identity of over 80% and a query coverage exceeding 95% (Supplementary Figure S2). The multiple sequence alignment showed that the functional motifs identified in BcStc3 are conserved, except in the XP_038737186.1 protein of *B. byssoidea*, which lacks the DDMWE motif due to an insertion of 30 amino acid residues at the first aspartic residue (Supplementary Figure S2). Additionally, the XP_038737186.1 protein was positioned on a distinct branch of the phylogenetic tree derived from the sequence alignment of the BcStc3 protein sequence with those of other *Botrytis* species, using the neighbor-joining method (Figure 2).

A second BlastP (https://blast.ncbi.nlm.nih.gov/Blast.cgi?PAGE=Proteins (accessed on 26 January 2024)) search was conducted, this time excluding the *Botrytis* and *Botrytioinia* taxids, and 168 homologous sequences were found according to Pearson et al. [45]. Five multidomain proteins (ESZ93961.1, KAH7563860.1, KAF4304151.1, EKG18134.1 and XP_049153155.1) and three other proteins (XP_051299547.1, CAI6339174.1 and KAG7009610.1), lacking the PF19086 domain or being partially annotated, were removed from the analysis. The 160 remaining sequences were distributed in 124 organisms distributed in Dikarya fungi (Supplementary Table S2). Seven proteins (1.5% of the total sequences) were annotated in three species of the Strophariaceae family (Agarycomycetes class, Basidiomycota), and the resting proteins were distributed in the Lectiomyceta (67.5%) and Sordariomyceta (28.1%) super-classes of Ascomycota (Table 2). Moreover, 60.6% of the total proteins were annotated in Eurotiomycetes and Dothideomycetes species (Table 2). The three motifs involved in catalysis were conserved in these proteins (Supplementary Figure S3). Noteworthy is the absence of BcStc3-like proteins in subphyla Taphrinomyctina and Saccharomycotina.

**Figure 3:**
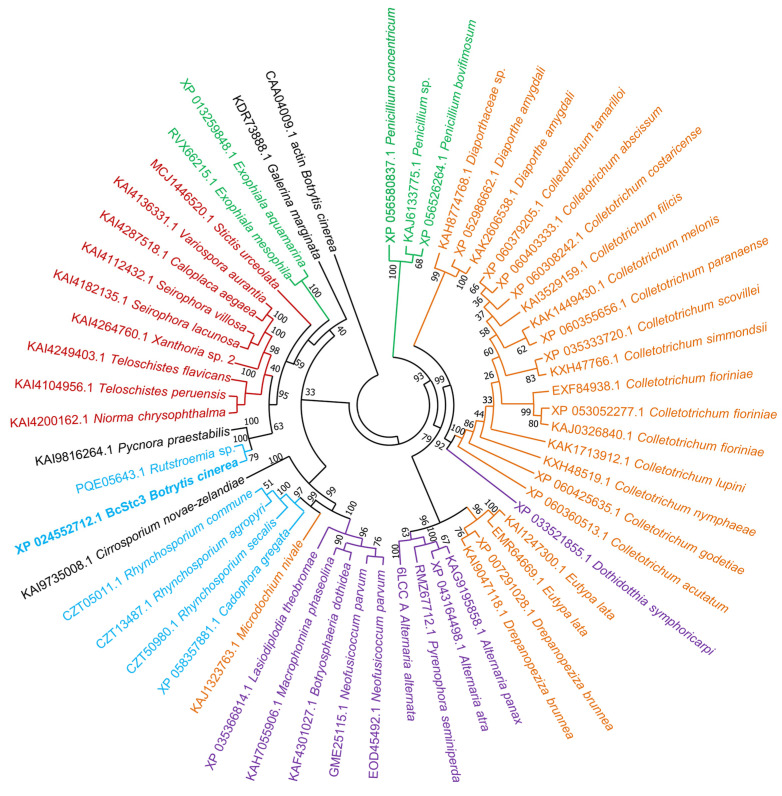
Phylogenetic tree of BcStc3 and homologous protein sequences of other fungi species. The phylogenetic tree was inferred using the neighbor-joining method via MEGA11 software (version v11.0) and bootstrap values from 1000 trials are indicated at each branch node. Protein sequences were selected after running similarity search by BlastP (https://blast.ncbi.nlm.nih.gov/Blast.cgi?PAGE=Proteins (accessed on 26 January 2024)) using BcStc3 as query sequence, excluding the *Botrytis* and *Botryotinia* taxids (33196 and 40558, respectively), and filtering the results based on percent identity >30%, coverage >70% and the bit-score >50. The top 55 hits were retrieved for sequence alignment and phylogenetic analysis. NCBI accession number of each sequence is shown. Actin (CAA04009.1) of *B. cinerea* was used as outgroup. Taxonomic distribution is highlighted by different colors: Eurotiomycetes (green), Sordariomycetes (orange), Dothideomycetes (violet), Leotiomycetes (blue) and Lecanoromycetes (red). The only members of the Xylobotryomycetes, Candelariomycetes and Agaricomycetes (Basidiomycota) classes are highlighted in black.

The alignment of the first 55 sequences retrieved from the BlastP (https://blast.ncbi.nlm.nih.gov/Blast.cgi?PAGE=Proteins (accessed on 26 January 2024)) search is used to construct a phylogenetic tree. Two clades are identified (Figure 3). Clade I includes most of the BcStc3-homologous proteins annotated in the Sordariomycetes fungi together with sequences from Eurotiales (Eurotiomycetes) and Pleorales (Dothideomycetes) organisms (Figure 3). Clade II consists of two groups. One is made up of BcStc3-related proteins from Botryosphaeriales (Dothideomycetes incertae sedis) and Helotiales (Leotiomycetes) fungi and the only member of the Xylobotryomycetes class (Figure 3). BcStc3 falls into the second group, clustered together with the PQE05643.1 protein of *Rutstroemia* sp. (Helotiales, Leotiomycetes) and the KAI9816264.1 protein of *Pycnora praestabilis*, the only member of the Candelariomycetes class (Figure 3). All the proteins of Lecanoromycetes fungi and one of the seven BcStc3-like proteins found in Basidiomycota (the KDR73888.1 protein of *Galerina marginata*) are also clustered in this second group (Figure 3).

### 2.2. Expression Analysis of the Sesquiterpene Cyclase Gene Family in B. cinerea

**Figure 4:**
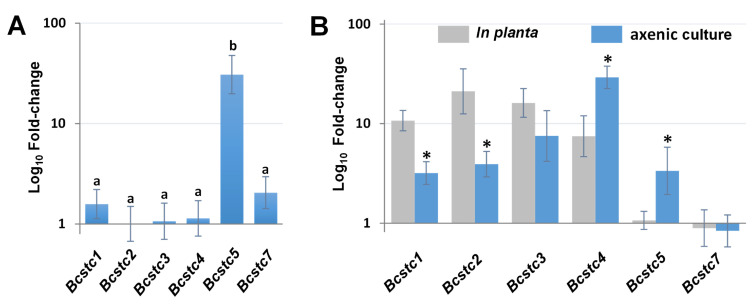
Gene expression of the *Bcstc* gene family in the *B. cinerea* UCA992 strain. (**A**) Relative expression levels in non-germinated conidia. mRNA levels were quantified by qRT-PCR, normalized to *actA* and *B-tub* mRNA levels, and expressed as a fold change over *Bcstc2* expression. (**B**) Relative expression levels in planta and axenic culture. mRNA levels were quantified by qRT-PCR in mycelium grown in YGG medium (axenic culture) and leaves of *N. tabaccum* infected with a conidial suspension (in planta). Values were normalized to *actA* and *B-tub* mRNA levels and expressed as a fold change over mRNA levels of each gene in non-germinated conidia. Error bars show standard deviation of three biological replicates (*n* = 3). Different letters above the bars in (**A**) mean that there are significant differences between n fold changes in the six genes (*p*-value < 0.05). * in (**B**) indicates significant differences between mRNA levels in in planta and axenic culture for each gene (*p*-value < 0.05).

The basal expression of the *Bcstc3* gene and the remaining members of the sesquiterpene cyclase gene family in the non-germinated conidia of the UCA992 strain was analyzed by qRT-PCR. The results were depicted as the fold change relative to the lowest expressed gene (*Bcstc2*). All family genes were expressed (Figure 4A). Differences between the relative expression of *Bcstc3*, *Bcstc1*, *Bcstc1*, *Bcstc7* and *Bcstc2* were not statistically significant, but the *Bcstc5* expression level was 30 times higher than the control (*Bcstc2*) (Figure 4A). The gene family was expressed differentially in the non-germinated conidia of the B05.10 strain. The *Bcstc1* mRNAs were the least abundant transcripts, and the expression levels of *Bcstc4* and *Bcstc7* were statistically similar to that of *Bcstc1* (Supplementary Figure S4A). However, the relative expression of *Bcstc2* and *Bcst3* was almost eight times higher than that of the *Bcstc1* gene, while the *Bcstc5* transcripts were the most abundant, up to 171 times higher when normalized against *Bcstc1* (Supplementary Figure S4A).

To evaluate the role of the sesquiterpene cyclases in the vegetative growth of the UCA992 strain, we compared the expression levels of the *Bcstc* family genes in mycelium after 96 h of axenic culture with the basal level of each gene in non-germinated conidia. All genes were upregulated except the *Bcstc7* gene, which hardly changed its expression (Figure 4B). mRNA abundances for *Bcstc3*, *Bcstc2*, *Bcstc5* and *Bcst1* increased from 7.5- to 3.3-fold, but *Bcstc4* was the most induced (29-fold) (Figure 4B). The transcription of all genes was enhanced in the B05.10 strain compared with the non-germinated conidia, with the *Bcstc7* gene showing the most significant upregulation (2000-fold) (Supplementary Figure S4B).

The *Bcstc3*, *Bcstc4*, *Bcstc1* and *Bcstc2* genes were overexpressed after 96 h of inoculating tobacco leaves with the conidia of the UCA992 strain (Figure 4B). The minimum and maximum n-fold changes relative to non-germinated conidia were 7.5 and 21 for the mRNA levels of *Bcstc4* and *Bcstc3*, respectively (Figure 4B). However, the *Bcstc5* and *Bcstc7* genes were not upregulated during the host–plant interaction (Figure 4B). On the contrary, the *Bcstc1* and *Bcstc7* genes were significantly overexpressed in planta (almost 700 times with respect to their basal level in non-germinated conidia) when tobacco leaves were inoculated with the B05.10 strain (Supplementary Figure S4B).

When comparing the deregulation of each gene in tobacco leaves and axenic culture, *Bcstc1* and *Bcstc2* were especially induced in tobacco leaves compared to the axenic culture despite being overexpressed in both conditions (Figure 4B). *Bcstc4* and *Bcstc5* exhibited higher overexpression in axenic culture, and *Bcstc3* was the only gene induced to the same extent in both tested conditions.

### 2.3. Co-Regulation of Sesquiterpene Cyclase Genes in B. cinerea

**Figure 5:**
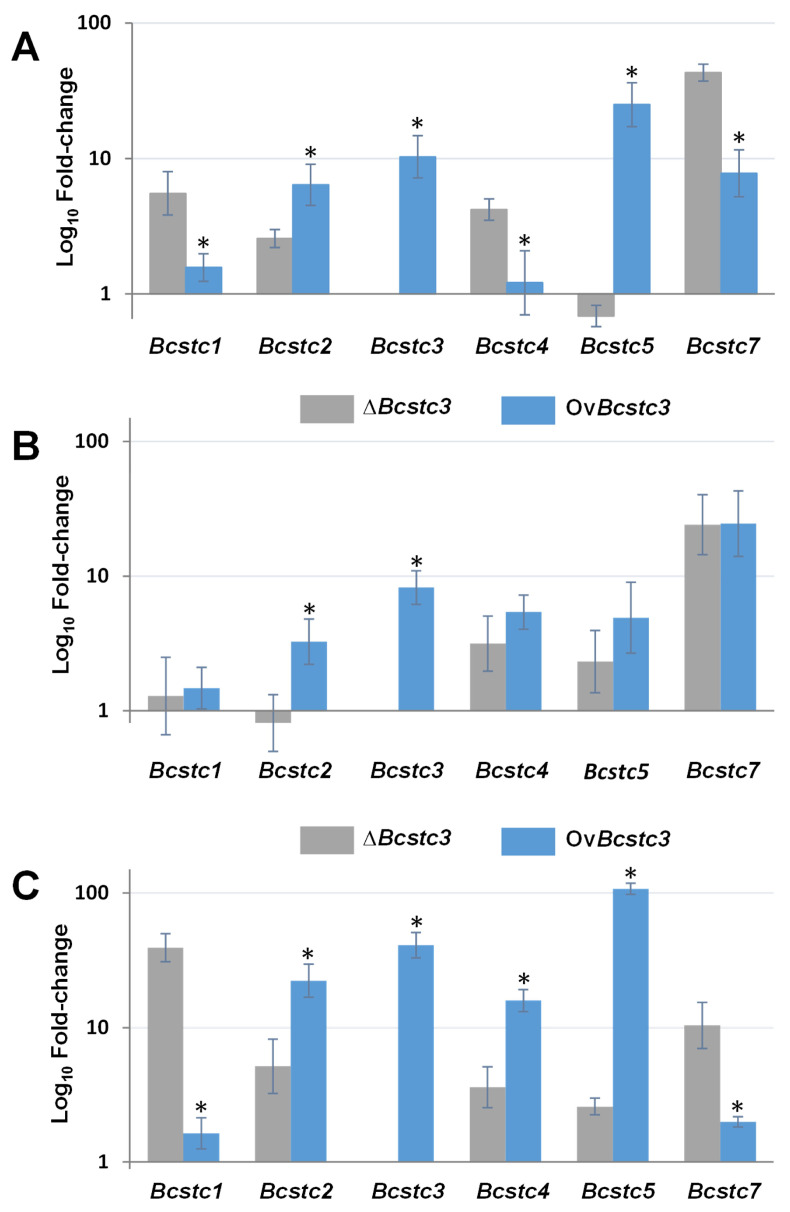
Gene expression of the *Bstc* gene family in the ∆*Bcstc3* and Ov*Bcstc3* strains. (**A**) Relative expression levels in non-germinated conidia. mRNA levels were quantified by qRT-PCR, normalized to *actA* and B-*tub* mRNA levels, and expressed as a fold change over mRNA levels of each gene in non-germinated conidia of the UCA992 strain, set as 1. (**B**) Relative expression levels in axenic culture. mRNA levels were quantified by qRT-PCR in mycelium grown in YGG medium for 96 h (axenic culture). Values were normalized to *actA* and *B-tub* mRNA levels and expressed as a fold change over mRNA levels of each gene in the UCA992 strain grown in the same conditions, set as 1. (**C**) Relative expression levels of the *Bcstc* genes in planta. mRNA levels were quantified by qRT-PCR in infected tissue of *N. tabaccum* leaves 96 h post inoculation with a conidial suspension of each strain. Values were normalized to *actA* and *B-tub* mRNA levels and expressed as a fold change over mRNA levels of each gene in the UCA992 strain under the same infection conditions. Error bars show standard deviation of three biological replicates (*n*  =  3). * means significant differences between the n-fold change in mRNA levels of each gene in each condition assessed in the ∆*Bcstc3* and Ov*Bcstc3* strains (*p*-value < 0.05).

The generation of the Δ*Bcstc3* strain and the overexpressed *Bcstc3* transformant (Ov*Bcstc3*) allowed us to analyze the possible co-regulation of the *Bcstc* gene family. In non-germinated conidia, the *Bcstc3* mutation induced the upregulation of the *Bcstc1*, *Bcstc2* and *Bcstc4* genes, but especially *Bcstc7* (over 40-fold changes), with respect to their expression levels in the conidia of the wild-type strain (Figure 5A). However, the relative mRNA transcript levels of *Bcstc5* did not change (Figure 5A). On the other hand, the conidia of the Ov*Bcstc3* strain overexpressed the *Bcsct5* gene (25-fold) and, to a lesser extent, the *Bcstc7* and *Bcstc2* genes (7.8- and 6.4-fold, respectively) with respect to the relative abundance of each transcript in the conidia of the wild-type strain (Figure 5A).

During fungal vegetative growth, the deletion and the overexpression of the *Bcstc3* gene also caused changes in the expression pattern of the gene family. After 96 h in axenic culture, the transcript levels of the *Bcstc4*, *Bcstc5* and especially *Bcstc7* (24-fold) genes were higher in the mycelia of the Δ*Bcsct3* and Ov*Bcstc3* strains than in the wild type (Figure 5B). However, the expression level of *Bcstc1* hardly changed, and the differential expression of *Bcstc2* was observed in both modified strains (Figure 5B).

Changes in the *Bcstc* gene family expression during the fungus–plant interaction were quite different in the *Bcsct3* knock-out and the *Bcsct3* overexpressing strains. The *Bcstc3* deletion induced the upregulation of the rest of the genes, especially *Bcstc1* and *Bcsct7* (increasing the transcripts levels to 40 and 10 times, respectively, related to the parental strain) (Figure 5C). However, the *Bcsct1* gene expression almost did not change in the Ov*Bcstc3* strain (Figure 5C). On the contrary, the overexpression of the *Bcstc3* gene caused a significant increase in the *Bcstc5* mRNA levels (up to 107 times related to the wild-type strain) while only 2.3 times in the mutant strain (Figure 5C).

When comparing the deregulation of each gene caused by *Bcstc3* deletion or overexpression in non-germinated conidia, *Bcstc1*, *Bcstc4* and *Bcstc7* were significantly more induced in the ∆*Bcstc3* mutant than in the Ov*Bcstc3* strain, and the contrary effect was observed for the *Bcstc4* and *Bcstc7* genes (Figure 5A). In vegetative mycelium, only *Bcstc2* showed a significantly higher overexpression in the Ov*Bcstc3* strain compared to the ∆*Bcstc3* mutant, while the remaining genes were induced to the same extent in both transformants (Figure 5B). Finally, in planta, *Bcstc1* and *Bcstc7* were significantly more induced in the ∆*Bcstc3* mutant, but *Bcstc2*, *Bcstc4* and *Bcstc5* were more induced in the Ov*Bcstc3* strain (Figure 5C).

### 2.4. BcStc3 Is Related to Fungal Growth and Stress Responses

**Figure 6:**
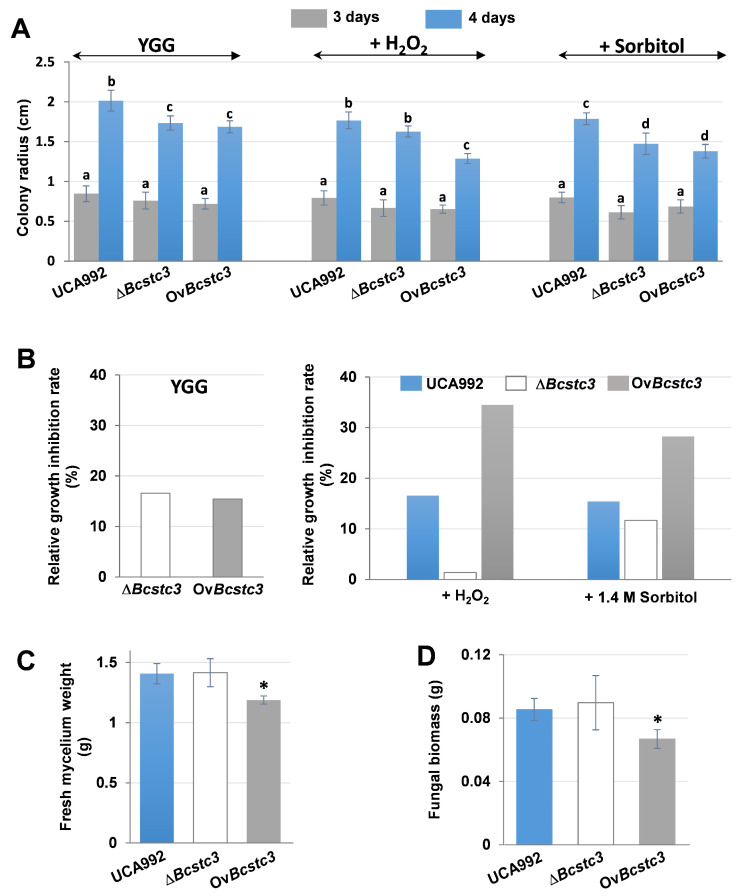
BcStc3 is involved in fungal growth and tolerance to stress agents. (**A**) Colony growth of the indicated fungal strains under different stressing conditions. It is represented the colony radius of the indicated strains grown in YGG medium (YGG) and YGG supplemented with 1.5 mM hydrogen peroxide (+H_2_O_2_) or 1.4 M sorbitol (+sorbitol) after 3 and 4 days of incubation at 20 °C. The results are presented as the mean value ± standard deviation of fifteen biological replicates (*n =* 15). (**B**) Relative growth inhibition of the transformants against the UC992 strain and of each fungal strain under different abiotic stresses. It is represented the relative growth inhibition rate of the Δ*Bcstc3* and Ov*Bcstc3* strains grown in YGG solid medium, with respect to the UCA992 strain (YGG), and the relative growth inhibition rates of the three fungal strains grown in YGG solid medium supplemented with 1.5 mM hydrogen peroxide (+H_2_O_2_) or 1.4 M sorbitol (+1.4 M sorbitol) compared to the growth of each strain in the same medium without the stress agents. (**C**) Fresh mycelium weight and (**D**) fungal biomass of the indicated strains after fungal growth in YGG liquid medium for 96 h at 20 °C (*n =* 3 biological replicates). Statistical significance is denoted by different letters above the bars in A (*p* < 0.05). * in (**C**,**D**) means statistical significance between UCA992 and Δ*Bcstc3* or Ov*Bcstc3* strains.

The potential role of BcStc3 in fungal development was investigated by comparing the growth of the Δ*Bcstc3* and Ov*Bcstc3* transformants with that of the wild-type strain in solid and liquid YGG media. After four days of growth on YGG agar, the colony radius of both transformants decreased by approximately 14% (Figure 6A), with similar results observed in the estimated growth rate of the colony radius (Figure 6B). In liquid culture, however, the mycelial fresh weight and fungal biomass were only reduced in the Ov*Bcstc3* strain, exhibiting decreases of 15% and 22%, respectively, compared to the UCA992 strain (Figure 6C,D).

The impact of deleting or constitutively expressing the *Bcstc3* gene on fungal tolerance to osmotic and oxidative stresses was investigated by cultivating the strains on YGG plates supplemented with 1.4 M sorbitol or 1.5 mM hydrogen peroxide. Compared to the wild type, the Δ*Bcstc3* strain exhibited a reduction in the colony radius of approximately 8% or 17% in media supplemented with peroxide or sorbitol, respectively (Figure 6A). However, both stress agents caused approximately a 25% reduction in the colony radius of the Ov*BcStc3* strain (Figure 6A).

The effect of sorbitol and hydrogen peroxide on the growth of each strain was evaluated by determining the relative growth inhibition rate. The results showed that hydrogen peroxide barely modified the growth rate of the ∆*Bcstc3* strain, while sorbitol led to a reduction of 11.6% compared to that in the absence of the stress agents (Figure 6B). The stressing conditions also affected the growth rate of the Ov*Bcstc3* transformant: osmotic or oxidative stresses reduced by 34.5% or 28.3%, respectively, in the growth rate of this strain relative to that of non-stressing conditions (Figure 6B). The relative growth inhibition rates of the UCA992 strain growing in hydrogen peroxide or sorbitol were 16.6% and 15.4%, respectively. The comparison of the values obtained for both transformants with those for the wild-type strain suggests that the deletion or overexpression of the *Bcstc3* gene modifies differentially the fungal ability to respond to stress conditions.

### 2.5. BcStc3 Is Involved in Virulence

**Figure 7:**
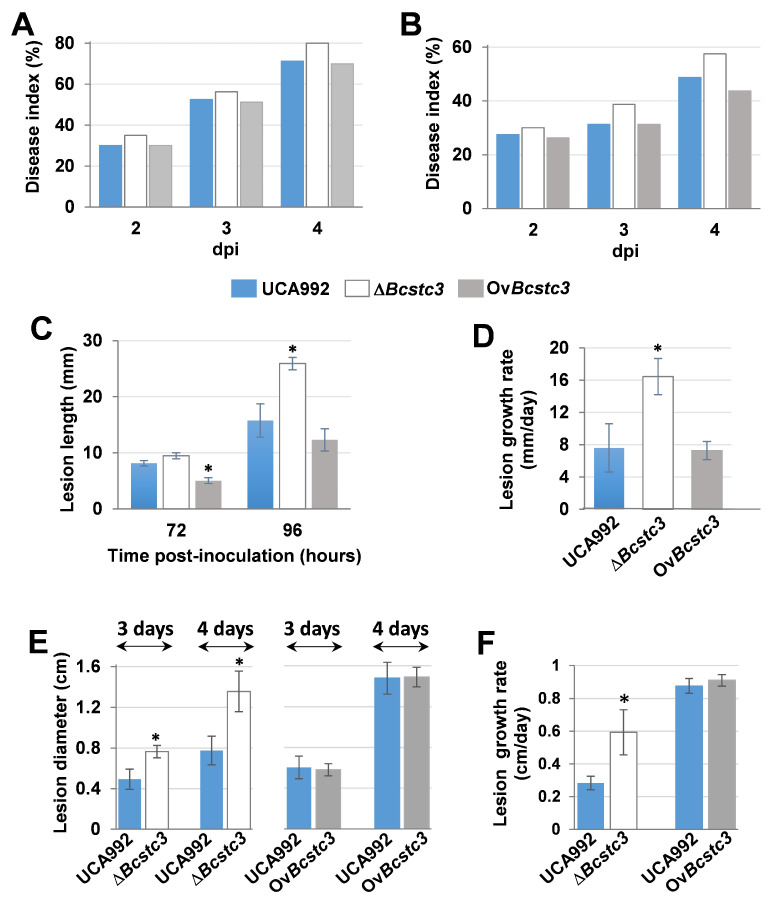
Deletion of the *Bcstc3* gene leads to increased virulence of *B. cinerea*. Different hosts were inoculated with 5 µL droplets containing 2500 conidia of the indicated strain in TGGK solution, incubated in the dark at 20 °C under high humidity conditions and the degree of damage was estimated. (**A**) Disease index (%) of tomato fruits at 2, 3 and 4 dpi estimated with a qualitative scale (Supplementary Figure S3A). (**B**) Disease index (%) of grapefruits at 2, 3 and 4 dpi estimated with a qualitative scale (Supplementary Figure S3). (**C**) Length and (**D**) growth rate (mm/day) of the lesions produced in gerbera petals estimated at days 3 and 4 after inoculation. (**E**) Diameter and (**F**) growth rate (cm/day) of the lesions produced in detached tobacco leaves 3 and 4 dpi. Fruit decay in tomatoes and grapefruits was determined for more than 30 fruits per fungal strain. The total number of inoculations on gerbera petals and tobacco leaves was greater than 15 per fungal strain and the results show the mean values ± SD. * means statistical significance between UCA992 and Δ*Bcstc3* or Ov*Bcstc3* strains.

To study the role of BcStc3 in fungal virulence, tomatoes, grapefruits, gerbera petals and detached tobacco leaves were inoculated with conidial suspensions of the wild-type, Δ*Bcstc3* and Ov*Bcstc3* strains. The Δ*Bcstc3* displayed increased virulence in all the tested hosts compared to the wild type. The disease index in tomatoes and grapefruits increased by 18% and 12%, respectively, compared to the wild-type strain at four days post inoculation (dpi) (Figure 7A,B, Supplementary Figure S5A,B). Also, the mean length of the lesions produced by the Δ*Bcstc3* on gerbera petals was 1.6-fold greater than those produced by the UCA992 strain at four dpi (Figure 7C, Supplementary Figure S5C), and the lesions grew two times faster compared to the control (Figure 7D). Finally, the mean diameter of the lesions caused by the mutant strain in tobacco leaves at four dpi and the lesion growth rate were also two times greater than those of the control (Figure 7E,F, Supplementary Figure S5D).

The disease index of Ov*Bstc3*-inoculated tomato fruits slightly and gradually decreased over time by 10.3% compared to the wild-type strain four days after inoculation (Figure 7A, Supplementary Figure S5A). However, there were no discernible differences between grapefruits infected by this transformant or the UCA992 strain (Figure 7B, Supplementary Figure S5B). The growth rate of the lesions produced by the Ov*Bcstc3* strain in gerbera petals was only 5% lower than the control, but the mean size of the lesions at 2 and 3 dpi were 38% and 22%, respectively, smaller than those produced by the wild-type strain (Figure 7C, Supplementary Figure S5C). On the other hand, no significant differences were found in the virulence of Ov*Bcstc3* and UCA992 strains when tobacco leaves were inoculated (Figure 7E,F, Supplementary Figure S5D).

### 2.6. BcStc3 Is Involved in the Germination and Morphology of Conidia

**Figure 8:**
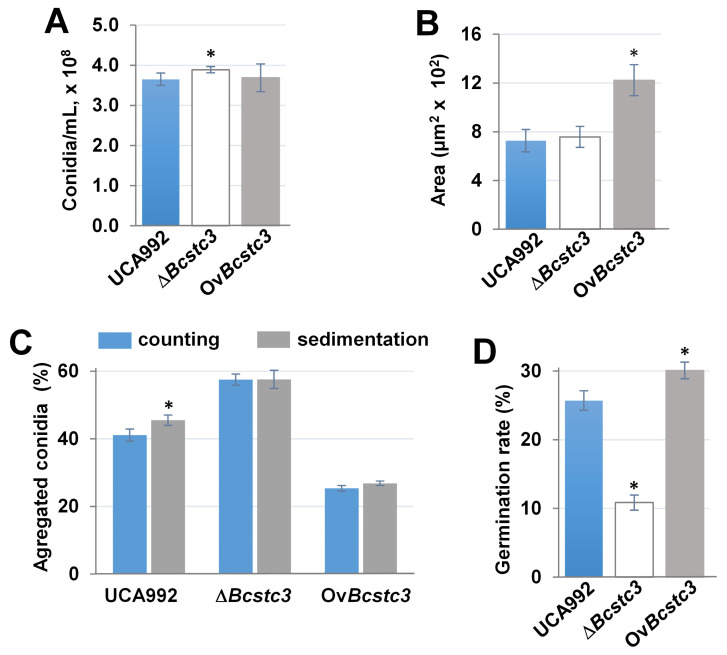
Involvement of BcStc3 in conidial morphogenesis and germination. (**A**) Conidial production of the indicated strain in tomato agar plates inoculated with agar plugs containing young mycelium exposed for 16 h to near-UV light for three days of inoculation and incubated at 20 °C for seven more days. (**B**) Bright-field pictures of conidia were taken with an Olympus BX-50 microscope and were used to measure the conidial area with the ImageJ software (version v1.54h, National Institutes of Health, USA) (*n* > 100). (**C**) The percentage of conidia clustered in groups of two or more was calculated from random bright-field pictures of 10,000 conidia in YGG incubated on a sterile glass slide for 6 h at 20 °C (counting), and the conidial sedimentation out of the suspension was estimated calculating the percentage of conidia that was sediment after 2 h at room temperature (sedimentation). (**D**) Conidial germination rate was calculated taking bright-field pictures of conidia incubated in YGG medium for 6 h with the microscope and counting conidia with elongating germ tube longer than the conidial longitudinal diameter (germinated conidia) and expressed as a percentage related to the total conidia counted (*n* > 100). * means statistical significance between UCA992 and Δ*Bcstc3* or Ov*Bcstc3* strains.

The production of conidia showed no significant difference between the control and transformants (Figure 8A). However, the conidial area of the Ov*Bcstc3* strain was almost twice that of the wild type (Figure 8B). The percentage of aggregated conidia increased by 38% in the conidia produced by the mutant and decreased by 38% in those produced by the Ov*Bcstc3* strain compared to the wild type (Figure 8C). Also, the opposite effect of deletion or constitutive expression of the *Bcstc3* gene on conidial germination was observed (Figure 8D). The conidial germination rate of the Δ*Bcstc3* strain decreased by more than 50% compared to the wild-type strain, while an increase of approximately 17% was observed for the Ov*Bcstc3* strain (Figure 8D).

### 2.7. Bcstc3 Gene Is Involved in ROS and Infection Cushion Production

**Figure 9:**
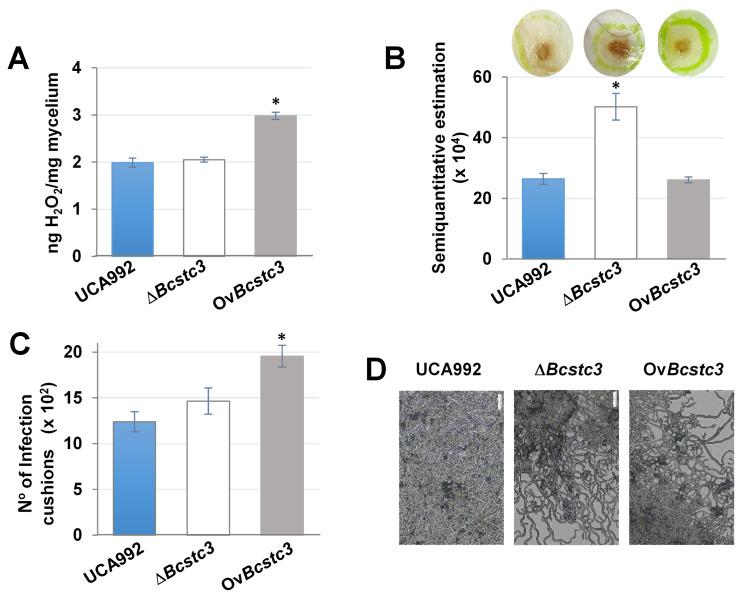
ROS production and BcStc3 are related. (**A**) ROS production in axenic culture by the indicated fungal strain incubating 24 mg of fresh mycelium with 1 mL of DAB solution for 5 h and measuring the absorbance at 417 nm after discarding the mycelium. (**B**) ROS production in the infected area of tobacco leaves by the indicated fungal strain 48 h after inoculation and vacuum-infiltrated for 1 h with 1 mg/mL DAB solution. ROS production was measured by ImageJ software (version v1.54h, National Institutes of Health, New York, NY, USA), and values were expressed as the percentage of brown pixels detected around the infection point. (Δ) Infection cushion production on one-quarter PDA plates after 48 h of incubation at 20 °C and quantified following the semiquantitative method described by [42]. (**D**) Pictures representative of the infection cushions of (**C**). * means statistical significance between UCA992 and Δ*Bcstc3* or Ov*Bcstc3* strains.

The BcStc3 protein seems involved in the fungal response to oxidative stress. Therefore, the production of reactive oxygen species (ROS) was studied under different growth conditions. In axenic culture, the Ov*Bcstc3* strain released nearly double the amount of ROS into the medium compared to the wild type, while the *Bcstc3* deletion did not affect the ROS generation (Figure 9A). In planta, the Δ*Bcstc3* strain produced nearly twice the amount of hydrogen peroxide in the lesion areas compared to the wild-type and Ov*Bcstc3* strains (Figure 9B).

On the other hand, as ROS is accumulated in the infection cushions [46], the production of these infection structures was assessed in the three fungal strains in axenic culture conditions. The results showed that the Ov*Bstc3* strain produced more infection cushions than the wild type, while no change was observed for the *Bcsct3* null mutant (Figure 9C).

### 2.8. Implication of the BcStc3 on the Secondary Metabolism

The polyketides and sesquiterpenes isolated from the culture media of the ∆*Bcstc3* and Ov*Bcstc3* strains were characterized by extensive spectroscopic and spectrometric mass analyses (Supplementary Figure S6). Their spectroscopic and physics constants were identical to those obtained from authentic samples isolated via OSMAC methodology from several previously reported *B. cinerea* strains [30,31,32,47,48,49].

The metabolomic analysis of the null mutant Δ*Bcstc3* broth yielded the polyketide derivatives **1** (1.8 mg), **2** (2.2 mg) and **3** (1.5 mg) [47], the known eremophil-9-en-11-ols **5** (6.5 mg), **6** (0.5 mg), **7** (3.0 mg), **8** (3.0 mg), **9** (1.3 mg), **10** (2.0 mg), **11** (2.8 mg), **12** (0.8 mg), **13** (0.8 mg) and **14** (0.7 mg) [31,48], the 11-hydroxyeremophil-1(10)-en-2-one **16** (0.5 mg) [30] and the 11,12,13-tri-*nor*-eremophilene **17** (3.8 mg) [32] (Supplementary Figure S6).

The overexpressed mutant Ov*Bcstc3* broth afforded the reported polyketide derivatives **2** (1.2 mg) and **4** (2.0 mg) and the known compounds **6** (3.4 mg), **7** (5.2 mg), **10** (1.5 mg), **11** (3.6 mg) [31,48], **15** (1.5 mg) [30], **17** (3.4 mg) and **18** (1.1 mg) [32] (Supplementary Figure S6).

Finally, the analysis of the metabolomic profile of both transformants, the null mutant Δ*Bcstc3* and the overexpressed Ov*Bcstc3*, seem to indicate that the production of secondary metabolites, as polyketides and sesquiterpenes, was not affected by mutations in *Bcstc3*. So, both transformants produced polyketide botcinins, as well as eremophilenol derivatives. Additionally, the Δ*Bcstc3* strain biosynthesized the keto derivative **16** and the 11,12,13-tri-*nor*-eremophilene (**17**), while the Ov*Bcstc3* transformant produced the keto derivative **15** and two 11,12,13-tri-*nor*-eremophilenes (**17**) and (**18**), all of them previously reported (Supplementary Figure S6) [30,31,32]. Unfortunately, no new sesquiterpenes were detected/produced by the overexpressed transformant.

## 3. Discussion

### 3.1. Bcstc3 Encodes for a Terpene Synthase Family 2, C-Terminal Metal-Binding Domain Protein

The *Bcstc3* gene encodes for a terpene synthase family 2, C-terminal metal-binding domain protein (Pfam 19086), according to the BLAST search against the NCBI non-redundant protein database, the same protein family of the pentalenene cyclase BcStc1/BcBot2 [27,28], the BcStc5 protein involved in the farnesyl diphosphate conversion into 2*Z*-4*E*-α-ionylideneethane [29] and the yet-to-be characterized BcStc4. The two remaining genes of the *Bcstc* family, *Bcstc2* and *Bcstc7*, encode for trichodiene synthases (Pfam 06330). Both enzyme families are members of the class I terpene synthase (cd00868, EC 4.2.3.-). BcStc3 showed at the C-terminal region the two motifs involved in the magnesium binding that are considered hallmarks in the class I terpene cyclases [9,10,11]. The aspartate-rich motif was identified at position 177 for the first aspartate residue and the NSE/DTE motif at position 307 for the asparagine residue (Supplementary Figures S1 and S3). Two other motifs involved in catalysis were also identified in the BcStc3 sequence: the conserved effector triad (R264-D267-I268 residues) that also coordinates the PPi, aiding in substrate recognition and is associated with the initiation of the cyclization reaction [9,10,40], and the conserved RY pair (R334-Y335 residues) (Supplementary Figures S1 and S3) that is involved in the PPi recognition through hydrogen bonds and the formation of the caryophyllenyl cation [40,49]. Two of the three residues involved in the selectivity of BcStc1/BcBot2 (W94, F99 and N325 residues) [47] were also identified in the sequence of BcStc3 (F174 and N392 residues) (Supplementary Figure S1).

The predicted BcStc3 three-dimensional model was an alpha-helix structure (Figure 1), characteristic of the class I terpene cyclase [37,38]. As expected, the DDMWE and NDLASYDKE motifs were identified at the entrance of the active site and the effector triad and the RY pair at the active site pocket (Figure 1). In the sesquiterpene cyclases BcStc1/BcBot2 and Cop3, Cop4, and Cop6 from *B. cinerea* and the basidiomycete *Coprinus cinereus*, respectively, the H-α1 loop moves and shields the active site upon the formation of an Mg^2+^3-PPi complex modulating the selectivity of these enzymes [42]. This structural motif was also identified in the BcStc3 3D-model, interacting with the conserved N329 residue at the C-terminus of the loop with an aspartic residue of the NSE/DTE motif to place the H-α1 loop at the catalytic site (Figure 1).

### 3.2. BcStc3 Is a Well-Conserved Protein in Botrytis Genus

The BcStc3 protein is a well-conserved protein in the *Botrytis* genus, especially in species that infect monocots Liliaceae (*B. tulipae*, *B. elliptica*), Amaryllidaceae (*B. sinoallii*, *Botryotinia globosa*, *B. byssoidea*, *Botryotinia narcissicola* and *B. galanthina*), Hyancinthacease (*B. hyacinthi*) and Asphodelaceae (*B. deweyae*) (Figure 2). The orthologous proteins of BcStc3 in *B. elliptica*, *B. hyacinthi*, *B. galanthina* and *B. narcissicola* had been previously identified [50], but this is the first time that homologous proteins to this sesquiterpene cyclase are identified in *B. sinoallii*, *B. tulipae*, *B. deweyae*, *B. byssoidea* and *Botryotinia globosa* (Figure 2, Supplementary Figure S2). The phylogenetic tree of these proteins closely resembled that previously described for *Botrytis* genes [51,52]: BcSct3 was grouped apart in clade I, while the remaining sequences formed clade II. Unexpectedly, the BcStc3-homologous protein of *B. byssoidea* (XP_038737186.1) was grouped apart (Figure 2), probably related to the lack of the aspartic-rich domain DDMWE (Supplementary Figure S2).

### 3.3. BcStc3 Is a Well-Conserved Protein in Kingdom Fungi

BcStc3 protein is also a well-conserved protein among the kingdom Fungi. Homologous proteins to BcStc3 were identified in 124 fungal species: seven proteins were found in three species of the Agarycomycetes class (Basidiomycota) and the remaining 153 proteins were distributed among 121 Ascomycota species (Table 2). The ratio of BcStc3-homologous proteins per organism was slightly higher in Basidiomycota than in Ascomycota which may be related to the described tandem duplication of the gene clusters in Basidiomycota [39,53,54]. Out of the 160 BcStc3 homologous proteins, only the terpene cyclase AaTPS of *Alternaria alternata* has been previously characterized [55]. AaTPS catalyzes terpene cyclization and switches to carry out aromatic prenylation reactions by alkaline conditions [55]. This bifunctional activity is well conserved among terpene cyclase family proteins [55]. Therefore, the BcStc3 protein may also function as an aromatic prenyltransferase in a pH-dependent manner.

BcStc3 is well conserved in the fungi of distantly related taxa. The closest protein to BcStc3 was the PQE05643.1 protein of *Rutstroemia* sp. (Figure 3), a phytopathogenic fungus of the Helotiales order that secretes diverse phytotoxic secondary metabolites involved in pathogenesis [56]. However, these two proteins formed a subclade with the KAI9816264.1 protein of *P. praestabilis* (Figure 3), a lichen fungus of the Candelariomycetes class known for having a small genome and a smaller number of secondary metabolite biosynthetic gene clusters [57,58]. Moreover, the three proteins clustered with homologous proteins of numerous lichenized Ascomycota, Basidiomycota and polymorphic black yeast-like fungi (Figure 3), all of them producers of diverse classes of secondary metabolites, some of which have clinical applications [56,59,60]. Over the past two decades, 122 sesquiterpene synthases (STSs) and two fusion enzymes have been unearthed and characterized from 26 basidiomycete species. These enzymes play a pivotal role in the biosynthesis of numerous sesquiterpenes through four distinct pathways [61]. Among the BcStc3 homologous proteins, seven sequences belonged to Basidiomycota fungi: the KDR73888.1 protein of *Galerina marginata* found among the first 55 sequences retrieved from the BlastP (https://blast.ncbi.nlm.nih.gov/Blast.cgi?PAGE=Proteins (accessed on 26 January 2024)) search, five other proteins in *Psilocybe cubensis* and one protein in *Psilocybe cyanescens* (Supplementary Information Table S2). The three fungal species belong to the order Agaricales, the largest mushroom-forming group, with over 400 genera and 13,000 species [62]. The three species contain in their genome more than 20 terpene biosynthetic clusters [59,63,64]. This high number of terpene biosynthetic gene clusters, often repeated in tandem, is a characteristic of Basidiomycota and is related to the fact that terpenes and terpenoids are produced more frequently by these fungi than by Ascomycota [53,64,65]. Horizontal gene transfer has been considered one of the primary mechanisms explaining the duplication of these clusters in Basidiomycota and also the transfer of genetic material between phylogenetically unrelated species [53,65,66]. For instance, the sesquiterpene cyclase involved in the synthesis of Δ6-protoilludene probably has been horizontally transferred from Basidiomycota to the Ascomycota *Diaporthe* sp. [65]. None of the BcStc3 homologous proteins found in Basidiomycota have been characterized. In *Galerina marginata*, only the sesquiterpene synthase *Galma_104215* has been described involved in the synthesis of β-gurjunene [59,64]; in *P. cubensis*, the sesquiterpene synthase CubA relates to the production of cubebol, β-copaene and various other sesquiterpenes or -terpenoids [63,67]; and in *P. cyanescens,* two sesquiterpene synthases involved in the synthesis of α-cuprenene have been described [63].

### 3.4. Bcstc3 Is Differentially Expressed during Fungal Development and Pathogenesis

The *Bcstc3* gene is not only expressed in non-germinated conidia but also upregulated up to 7 and 16 times during fungal vegetative growth and plant–host interactions, respectively, compared to the expression level in non-germinated conidia (Figure 4). This expression pattern, however, differs from that in the B05.10 strain (Supplementary Figure S4). Variations between strains had also been found when grown in sublethal levels of copper sulfate [48]. These results suggest a potential role of BcStc3 in the biology of *B. cinerea* in a strain-dependent manner.

So far, the expression of the *Bcstc* gene family has been studied under specific growth conditions (supplementing the culture medium with copper sulfate or after long-standing growth culture [29,30,31,32,48]). Therefore, this is the first time that the expression of the six members of the *Bsctc* gene family has been shown in non-germinated conidia. These data enabled, also for the first time, the analysis of the gene expression changes induced during fungal vegetative development and the interaction with the plant host. Furthermore, the comparison between the expression patterns in different strains revealed a differential regulation in a fungal strain-dependent manner, suggesting a functional specialization through differential expression patterns.

The generation of two new strains, the ∆*Bcstc3* and Ov*Bcstc3* transformants, allowed us to study the putative effect of the *Bcstc3* deletion or overexpression, respectively, on the regulation of the *Bcstc* gene family in *B. cinerea* (Figure 5). In non-germinated conidia, the deletion of the *Bcstc3* gene caused the down-regulation of *Bcsct5*, but the contrary effect was observed when the *Bcstc3* gene was overexpressed (Figure 5). During the vegetative growth of the ∆*Bcstc3* mutant, the expression of the *Bcstc2* gene was down-regulated, while its relative expression increased in the Ov*Bcst3* strain (Figure 5). All *Bcstc* genes were overexpressed in both transformants during the fungal–host interaction, although with differential gene expression patterns, with *Bcsctc1* and *Bcstc5* being the most upregulated in the ∆*Bcstc3* and Ov*Bcstc3* strains, respectively (Figure 5). These results show, for the first time, the differential crosstalk in the gene expression of the *Bcstc* gene family not only in conidia but also during vegetative growth and plant interaction.

### 3.5. BcStc3 Is Involved in Fungal Development and Tolerance to Osmotic and Oxidative Stress

The putative role of the BcStc3 protein in the biology of *Botrytis* was ruled out by studying the fungal phenotypic changes due to the deletion or the constitutive expression of the *Bcstc3* gene in the Δ*Bcstc3* and Ov*Bcstc3* strains, respectively. The growth rate of both strains was reduced by 14% compared to the wild type on solid medium, but only the biomass of the Ov*Bcstc3* strain decreased when cultured on a liquid medium (Figure 6). These results suggest that the BcStc3 overproduction affected negatively the fungal vegetative growth, while its lack only reduced the ability to grow on solid medium. The differential expression pattern of the remaining *Bcstc* genes in both strains found during axenic culture (Figure 5) may also have a potential effect on these results. The role of *Bcstc7* and *Bcstc1*/*Bcbot2* in mycelial development has already been ruled out [28,30], but it is yet to be established whether *Bcstc2*, *Bcstc4* or *Bcstc5* are involved in fungal growth. In fact, the differential deregulation of *Bcstc2* and fungal biomass production observed in the ∆*Bcstc3* and Ov*Bcstc3* strains may be correlated (Figure 6). The specific role of BcStc3 in the vegetative growth of *B. cinerea* remains to be elucidated.

A strong correlation has beIn identified between the regulation of secondary metabolism and stress triggers, modulating environmental adaptation and stress tolerance in filamentous fungi [68,69,70,71]. Therefore, the putative role of BcStc3 to overwhelm stress conditions was investigated by assessing the effect of sorbitol and hydrogen peroxide on mycelium development. The deletion of the *Bcstc3* gene slightly reduced the sensitivity to sorbitol, while the BcStc3 overproduction doubled the sensitivity to the osmotic agent (Figure 6). Therefore, the secondary metabolites generated as end products of the pathway in which BcStc3 is involved may affect the ability of *B. cinerea* to respond effectively to osmotic stress conditions. Furthermore, the enhanced sensitivity to osmotic stress and the reduced fungal biomass (Figure 6) of the Ov*Bcst3* strain may be related.

The overproduction of BcStc3 also increased sensitivity to oxidative stress, but the lack of the protein almost induced total resistance to these stress conditions (Figure 6). The accumulation of the secondary metabolites related to BcStc3 enzymatic activity may trigger ROS production or alternative metabolic pathways that alter the redox balance within the cell. The terpenoid kojic acid produced by *Aspergillus flavus* acts through free radical scavenging mechanisms, but its overproduction causes iron deficiencies that affect the regulation of siderophore biosynthetic genes and other signalling pathways [72]. In the basidiomycete *Ganoderma lucidum*, the reduced production of the triterpenoid ganoderic acid increased fungal resistance to oxidative stress (increasing intracellular ROS levels and inhibiting the enzymatic activity of the antioxidant systems) [73]. On the other hand, the constitutive expression of *Bsct3* also induced an enhanced production of ROS in the Ov*Bcstc3* strain in axenic culture (Figure 9) that may be related to the hypersensitivity of this strain to (additional) exogenously applied oxidative stresses (Figure 6). On the contrary, the lack of BcStc3 did not change ROS production (Figure 9). These results support the idea that BcStc3 is involved in fungal tolerance to oxidative stress but not related to ROS production.

### 3.6. BcStc3 Is Involved in Conidial Morphogenesis and Infection Cushion Production

Secondary metabolism gene clusters exhibit increasingly dynamic and differential expression during asexual growth, conidiation, and sexual development in filamentous fungi [74,75]. The BcStc3 protein did not seem to be related to the production of conidia since the deletion or overexpression of the *Bcstc3* gene did not cause any change in the ability to produce these asexual reproductive structures (Figure 8). However, BcStc3 played a role in conidial morphogenesis as the size and possibly the composition/structure of the surface of conidia changed when the BcStc3 protein was overproduced (Figure 8). Recently, it has been described that the polyketide synthase PKS15 of the entomopathogenic fungus *Beauveria bassiana* may act in cellular wall formation, and its metabolite could directly be a spore wall component [76]. The BcStc3 protein is also involved in conidial germination. Its deficiency resulted in a 50% reduction in the germination rate, while its overproduction led to a 17% increase compared to the wild type (Figure 8). The role of other members of the *Bcstc* gene family in conidiogenesis has been partially characterized. The BcStc1/Bcbot2 protein involved in botrydial biosynthesis did not affect conidial morphogenesis [28]. The deletion of the *Bcstc5*/*Bcaba5* gene did not induce any change in the conidiation of the ABA-overproducing strain ATCC58025, which is known to be deficient in conidia and sclerotia formation [29]. BcStc7 catalyzes (+)-4-epi-eremophil-9-en-11-ols biosynthesis that enhances the conidiophores’ production when added externally to the *Botrytis* culture [30]. The different expression patterns of the *Bcstc* genes found in the ∆*Bcstc3* and Ov*Bcstc3* strains likely also contribute to the variations observed in the germination rate, conidial size and aggregation between both transformants (Figure 8).

The infection cushions are specialized structures produced by certain pathogenic fungi formed by the aggregation of fungal hyphae and specialized cells at the site of infection, promoting the penetration and colonization of the host tissues [77,78]. Here, we show that BcStc3 is also involved in the generation of these structures since the overproduction of BcStc3 led to an almost twofold rise in the infection cushion number compared to those produced in axenic culture by the wild-type and mutant strains (Figure 9). This finding may be related to the enhanced production of hydrogen peroxide detected in the OvBcStc3 strain (Figure 9) as the infection cushions serve as physical barriers that shield the fungal hyphae from the damaging effects of ROS produced by the host plant, displaying mechanisms such as the upregulation of antioxidant enzymes or the synthesis of protective metabolites [46]. The *Bcstc1*/*Bcbot2* and *Bcboa6* genes encoding for the core enzymes of the BOT and BOA biosynthetic gene clusters, respectively, are also upregulated in these infective structures [46], and the sesquiterpenoid eremophilenols, whose synthesis involves the sesquiterpene cyclase BcSct7, may act as endogenous signals to promote and regulate the generation of infection cushions [30].

### 3.7. BcStc3 in Involved in Virulence

Unexpectedly, the Δ*Bcstc3* strain exhibited a higher infection efficiency than the wild type in all hosts tested, and the *Bcstc3* overexpression only caused a slight decrease in the disease index in tomato fruits compared to that of the UCA992 strain (Figure 7).

Why does the deletion of the *Bcst3* gene increase fungal virulence? In this work, we observed that the ROS production at the inoculation site in tobacco leaves was twice as high whether the conidia of the ∆*Bcstc3* strain were used instead of those of the wild-type or Ov*Bcstc3* strains (Figure 9). On the other hand, the *Bcstc3* deletion induced significant changes in the expression pattern of the remaining *Bcstc* genes, particularly the upregulation of *Bcstc1*/*Bcbot2* (Figure 5). BcSct1/BcBot2 is involved in the synthesis of botrydial, a sesquiterpene that triggers the hypersensitive response on plant tissue, inducing the accumulation of ROS and phenolic compounds and causing chlorosis and cell collapse [22]. The overexpression of the *Bcstc1*/*Bcbot2* gene during the infection of tobacco leaves by the ∆*Bcstc3* transformant may be related to the increased production of ROS in the host interaction and the enhanced virulence of the ∆*Bcstc3* strain. The lack of the BcStc3 protein and the BcStc1/BcBot2 overproduction may modify the oxidative balance at the infection site, increasing the fungal capability to penetrate and invade host plant tissues. Similar results were found in the null mutant of the *bcpks13* encoding for a polyketide synthase involved in melanin synthesis. The mutant strain caused larger lesions and accumulated ROS more rapidly and abundantly than the wild type at the infection site by inducing much stronger plant responses [79].

The upregulation of *Bcsct7* in the ∆*Bcstc3* strain in planta may also be related to the increased virulence of this strain (Figure 5). BcStc7 catalyzes the synthesis of eremophilenols with an underlying carbon skeleton which is enantiomeric to that of capsidiol, a phytoalexin produced by green peppers when they are infected by phytopathogenic fungi [30,31]. This structural similarity between capsidiol and eremophilenols suggests that fungal secondary metabolites may modulate the plant defense response to facilitate the attack of the plant tissue [30,31].

On the other hand, the activity of many secondary metabolites as effectors that contribute to pathogen virulence is well known [80,81,82]. It cannot be ruled out that the alterations of the pathway in which *Bcstc3* participates might cause the biosynthesis of other secondary metabolites that could act as effectors, increasing the infective capacity of the fungus and/or modulating the plant defense response.

## 4. Materials and Methods

### 4.1. Bioinformatic Analysis

The BcStc3 sequence was used as a query to perform a BlastP (https://blast.ncbi.nlm.nih.gov/Blast.cgi?PAGE=Proteins (accessed on 26 January 2024)) search against the non-redundant protein sequence database at the National Centre for Biotechnology Information (NCBI) [83]. Homologous proteins were selected from the resulting list following the criteria: percent identity (>30%), coverage (>70%) and the bit-score (>50) [45]. Sequence identities were calculated by the Sequence Identity And Similarity (SIAS) module from (http://imed.med.ucm.es/Tools/sias.html (accessed on 22 January 2024)] [84]. Phylogenetic analyses were performed by the neighbor-joining method with 1000 bootstrap replicates using the MEGA11 software (version v11.0) [85]. The predicted BcStc3 3-D model was obtained from the Alphafold tool (https://alphafold.ebi.ac.uk/ (accessed on 8 December 2023), (version v2.1.2) [41,86], using the default parameters (https://github.com/google-deepmind/alphafold (accessed on 8 December 2023)). The resulting protein structure was then visualized with the PyMOL Molecular Graphics System (Version 1.8, Schrödinger, LLC, New York, NY, USA).

### 4.2. Organisms, Media and Culture Conditions

The *Bcstc3* knock-out mutant and the Ov*Bcstc3* strain were generated in this work from the parental *B. cinerea* strain UCA992, obtained from the Domecq vineyard (Jerez de la Frontera, Cádiz, Spain) and deposited in the Mycological Herbarium Collection of the University of Cádiz. All fungal strains were routinely grown in YGG medium (2% glucose, 0.5% yeast extract, 0.3% Gamborg’s B5 medium (Duchefa Biochemie) and 1.5% agar, when needed) at 20 °C for three days. Tomato agar plates (25% homogenized tomato fruits (w/v), 1.5% agar, pH 5.5) were used for conidia production. Potato dextrose agar (PDA) (Sigma-Aldrich, St. Louis, MO, USA) medium (1/4) was used to estimate the infection cushion production. The conidial stock suspensions were maintained in 10% glycerol at −80 °C. For DNA extraction from mycelium, fungal strains were incubated in Petri dishes containing completed medium (CM) (10 g glucose, 2 g casein peptone, 1 g casamino acids, 1 g yeast extract, 50 mL salt solution, 1 mL vitamin solution (0.5 g biotin, 50 g nicotinamide, 16 g *p*-aminobenzoic acid and 20 g pyridoxine hydrochloride per liter) and 2 mL microelement solution (1 g FeSO_4_·7H_2_O, 0.15 g CuSO_4_·5H_2_O, 1.61 g ZnSO_4_·7H_2_O, 0.1 g MnSO_4_·H_2_O, 0.1 g (NH_4_)_6_Mo_7_O_24_·4H_2_O per liter) per liter, pH 5.0) overlaid with cellophane for 3 to 4 days at 20 °C.

For the *S. cerevisiae* strain FY834 [30,87] transformation, plates of SD-uracil medium (D-glucose 20 g, Difco^®^ (Montreal, CA, USA) yeast nitrogen base without amino acids 6.7 g, Clontech –Ura DO supplement 0.77 g, agar 16 g per liter, pH 5.8) were used, and they were incubated for 3 or 4 days at 30 °C.

*N. tabacum* var. Havana plants were maintained in a growth chamber at 22 °C and 70% humidity under a light/dark cycle of 14 h light/10 h dark. *Gerbera jamesonii*, grapes and tomato fruits were purchased from local groceries.

### 4.3. Standard Molecular Methods for Gene Inactivation and Overexpression

Fungal genomic DNA was isolated following the protocol of Mansfield (1985) [88]. Bacterial plasmids were isolated using the GeneJET PCR Plasmid Purification Kit (Thermo Scientific, Waltham, MA, USA). A NanoDrop 2000c spectrophotometer (Thermo-Scientific) was used for checking the purity and concentration of the DNA samples. Phusion High-Fidelity DNA Polymerase (Thermo-Scientific) and Go-Taq DNA Polymerase (Promega, Madison, WI, USA) were used for conventional PCR. Agarose gel electrophoresis and restriction enzyme digestions were performed using standard procedures [89]. For the sequence analysis, DNASTAR Lasergene package (DNASTAR, Inc., version 17.6) programs were used. Oligonucleotides (Supplementary Table S1) were from Metabion International AG.

### 4.4. Generation of the ΔBcstc3 and OvBcstc3 Strains

For the generation of *Bcstc3* (Bcin13g05830) deletion mutants, two regions flanking the *Bcstc3* ORF of 910 bp and 630 bp were amplified using primer pairs *Bcstc3*-5F/-5R and *Bcstc3*-3F/-3R, respectively (Supplementary Table S1). The two DNA fragments were assembled with a hygromycin resistance cassette obtained by PCR using plasmid pCSN44 as the template and primer pair *Hph*-F/-R and with the *EcoR*I/*Xho*I-digested plasmid pRS426 [90,91] by yeast recombinational cloning using the strain *S. cerevisiae* FY834 [30,87]. The resulting *Bcstc3* replacement fragment (3029 bp) was amplified with primer pair *Bcstc3*-5F and *Bcstc3*-3R and used to transform the protoplasts of the *B. cinerea* UCA992 strain generated as described previously by Schumacher (2012). Transformants were selected by hygromycin resistance [90]. Homokaryotic mutant strains were generated using a single-spore isolation protocol as described by Gonzalez-Rodriguez et al. [87] and Schumacher [90] and confirmed by PCR amplification with the primer pairs *Bcstc3*-WT-F and *Bcstc3*-WT-R (Supplementary Table S1). To confirm the homologous integration of the replacement cassette at the *Bcstc3* locus, fungal DNA was isolated and amplified with the primer pairs *Bcstc3*-hi5F/*TrpC*-T and *TrpC*-P2/*Bcstc3*-hi3R to detect targeted integration at 5′ or 3′ ends, respectively (Supplementary Table S1).

For the generation of the *Bcstc*3-overexpressed strain (Ov*Bcstc3*), the ORF of *Bcstc3* (1758 bp) was amplified using primers Tt*rpC*-*Bcstc*3-F and P*actA*-*Bcstc*3-R with DNA from the parental strain UCA992 serving as the template. The amplicon was then assembled with a fragment containing the *actA* promoter (P*actA*), obtained by PCR using primers P*actA*-amp-F and P*actA*-P*trpC*-R (Supplementary Table S1), and the plasmid pNDN-AGT [68] as the template, followed by digestion with the *Spe*I and *Not*I enzymes of the same plasmid in yeast FY834 [30,87], resulting in the vector pNDN-*Bcstc*3-OVER. This resulting vector contains the ORF *Bcstc3* gene under the control of the *actA* gene promoter and the *trpC* terminator (TtrpC) from *A. nidulans* [67], along with a nourseothricin resistance cassette (PtrpC::nat1), flanked by fragments of the *B. cinerea Bcnia*D gene, encoding nitrate reductase. Protoplasts of the UCA992 strain were transformed with the *Bcstc*3Ov replacement fragment (6400 bp), obtained by PCR amplification using the primer pairs BcniaD-5F and BcniaD-3R (Supplementary Table S1) and vector pNDN-*Bcstc*3-OVER as the template. Transformants were selected for nourseothricin resistance, and heterokaryons were generated. The targeted integration of the *Bcstc3* expression cassette at the *Bcnia*D locus was checked by PCR with primer pairs BcniaD-hi5F/PactA-stc3-R and BcniaD-hi3R/PactA-amp-F, and heterokaryotic mutants were confirmed by PCR amplification with the primer pairs BcniaD-WT-F and BcniaD-WT-R (Supplementary Table S1).

### 4.5. Quantitative Assessment of Gene Expression via qRT-PCR

The fungal mycelium from *B. cinerea* UCA992 wt, Δ*Bcstc3*, Ov*Bcstc3* and *B. cinerea* B05.10 wt used for total RNA extraction was collected by filtration from 30 mL of YGG medium inoculated with 5 µL of 105 conidia/mL and incubated at 20 °C for four days in darkness. To study in planta gene expression, the detached leaves of *N. tabacum* var. Havana plants were inoculated with 5 µL droplets of a conidial suspension of 10^5^ conidia/mL in TGGK solution (60 mM KH_2_PO_4_, 10 mM glycine, 0.01% Tween 20, 100 mM glucose) of *B. cinerea* UCA992 wt, Δ*Bcstc3*, Ov*Bcstc3* and *B. cinerea* B05.10 wt and incubated for 96 h at 22 °C in darkness with 70% humidity. The infected areas were cut and frozen at −80 °C until use. Mycelia and infected plant tissues were homogenized with a drill, and total RNA was extracted using the Trizol reagent (Sigma-Aldrich, T 9424), following the manufacturer’s instructions.

cDNA synthesis from 1 µg of total RNA was performed using the iScript cDNA Synthesis Kit (Bio-Rad, Hercules, CA, USA) according to the manufacturer’s instructions. qRT-PCR was carried out on an iCycler iQ system (Bio-Rad, USA) using the iQ SyBR Green Supermix (Bio-Rad, USA) and the primers listed in Supplementary Table S1. The *actA* (Bcin16g02020) and *B-tub* (Bcin01g08040) genes of *B. cinerea*, encoding for actin and tubulin, respectively, were used as internal controls to normalize the cDNA samples. The relative amount of mRNA was calculated by the ΔΔ*Ct* method [92] for the mean of three independent determinations of the threshold cycle (Ct). The results were represented as mean values of 2^−(ΔΔCt ±SD)^ (fold value) normalized to the expression of each gene in non-germinated conidia, vegetative mycelium or during tobacco leaf infection in the UCA992 strain (or the B05.10 strain), depending on the conditions tested.

### 4.6. Phenotypic Characterization of Fungal Transformants

#### 4.6.1. Vegetative Growth and Tolerance to Stress Agents

Petri dishes containing YGG-agar medium were inoculated with 10 µL droplets of a conidial suspension of 5 × 10^6^ conidia/mL and incubated at 20 °C to test for altered fungal growth. The colony radii were measured daily for four days, and the radial growth rate was calculated by plotting the colony radius over time, which was fitted to a linear model (Pearson’s correlation coefficient value (r^2^ ≥ 0.98)). The results are presented as the mean values of the growth rates in cm/day ± standard deviation of at least six independent experiments. To assess the sensitivity of fungal strains to stress conditions, the YGG-agar medium was supplemented with 1.4 M Sorbitol (S1876-1; Sigma-Aldrich; USA) or 1.5 mM H_2_O_2_ (018556; Foret; Barcelona, Spain) for osmotic or oxidative stress, respectively. The relative growth inhibition rate was calculated as follows: Inhibition rate(%) = [1 − (T_4_ − T_3_)/(C_4_ − C_3_)] × 100, where T and C represent the radius of the colonies (cm) in treated and control plates, respectively, and 4 and 3 the days of incubation at 20 °C [93].

In order to evaluate fungal biomass accumulation in liquid culture, 30 mL of YGG medium was inoculated with 10 µL of a conidial suspension of 5 × 10^6^ conidia/mL and incubated at 20 °C without shaking for four days. Mycelia were harvested by filtration, washed with sterile water, and dried for 1 h at room temperature to obtain the fresh weight. The dry weight was estimated by incubating the mycelium at 50 °C until a constant weight was achieved (dry weight). The experiment was conducted in triplicate, and the results are presented as the mean values of the biomass (g) ± standard deviation.

#### 4.6.2. Conidial Production and Germination

Tomato agar plates (25% homogenized tomato fruits (*w/v*), 1.5% agar, pH 5.5) were inoculated with agar plugs containing young mycelium (0.5-cm YGG-agar cubes), incubated for three days at 20 °C in dark conditions and exposed for 16 h to near-UV light at a distance of 30–40 cm. After seven more days in darkness, conidia were collected as described by van der Vlugt-Bergmans et al. [89] and quantified using a hemocytometer under a bright-field microscope Olympus BX-50 (Olympus Life Science, Waltham, MA, USA). The results are presented as the mean values ± standard deviation of six individual experiments.

To assess the conidial germination rate, aliquots of 20 µL of a 10^5^ conidia/mL suspension in YGG medium were placed on a sterile glass slide and incubated at 20 °C in the dark and under humidity conditions. After 6 h, bright-field pictures of conidia were taken with an Olympus BX-50 microscope, and in each field examined, at least 100 conidia were counted. Conidia were considered germinated when the elongating germ tube was longer than the conidial longitudinal diameter. Conidial germination rate values are expressed as the percentage of germinated conidia to the total conidia counted.

To study conidial aggregation, the percentage of conidia clustered in groups of two or more was calculated from random bright-field pictures of 10,000 conidia in YGG incubated on a sterile glass slide for 6 h at 20 °C in high humidity conditions. On the other hand, conidial aggregation was also estimated from the conidial sedimentation out of the suspension. The optical density of a 10^8^ conidia/mL suspension in water was measured at 600 nm and of the remaining suspended conidia after 2 h at room temperature. The optical density values were used to estimate the conidial concentration using a standard curve prepared from dilutions of a conidial suspension of a known concentration. The conidial aggregation was calculated using the following equation: % aggregated conidia = 100 × ((initial conidia concentration − final conidia concentration)/initial conidia concentration).

#### 4.6.3. Virulence Assays

Detached tobacco leaves, gerbera petals and tomatoes and grapefruits were inoculated with 5 µL droplets containing 2500 conidia in TGGK solution (60 mM KH2PO4, 10 mM glycine, 0.01% Tween 20 and 100 mM glucose), incubated in the dark at 20 °C under conditions of high humidity and photographed every 24 h. The diameter and length of the lesions on the tobacco leaves and gerbera petals, respectively, were measured using ImageJ software (version v1.54h, National Institutes of Health, USA) [94], and the lesion growth rate was calculated and expressed in cm/day or mm/day, as indicated in Figure 8.

A semiquantitative scale with four grades according to the severity of disease symptoms was used to evaluate the virulence of the fungus in fruits. The results are presented as the percentage of fruits in each disease grade to the total number of infected fruits (>30). Disease scores from 0 to 4 were subsequently converted to a percentage fruit disease index (%DI) as follows: [∑(disease rank × number of fruits)/(highest disease rank × total number of fruits)] × 100.

#### 4.6.4. Reactive Oxygen Species and Infection Cushion Production

The quantitative determination of H_2_O_2_ was performed incubating 24 mg of fresh mycelium with 1 mL of DAB solution for 5 h at room temperature. The mycelium was harvested, and the optical density of the supernatant was measured at 471 nm. The absorbance values were compared with a standard curve prepared with known peroxide concentrations. The results were presented as ng of H_2_O_2_/mg of mycelium.

In vivo ROS was analyzed in tobacco leaves inoculated with 5 µL droplets of a 5 × 10^5^ conidia/mL conidial suspension in TGGK solution and incubated for 48 h at 20 °C. Leaf discs, including the infected area, were cut and vacuum-infiltrated for 1 h with 1 mg/mL of DAB solution, pH 3.7. Discs were boiled in ethanol for 10 min to eliminate chlorophyll and photographed. ROS production was measured by ImageJ software (version v1.54h, National Institutes of Health, New York, NY, USA), and values were expressed as the percentage of brown pixels detected around the infection point.

The production of infection cushions was assessed following the semiquantitative method described by Sekulska-Nalewajko et al. in 2016 [95].

### 4.7. Statistical Analysis

Statistical analysis was performed with the SPSS (IBM) software package (version 24.0). The normal distribution of data was analyzed with the Kolmogorov–Smirnov (N > 50 samples) or Shapiro–Wilks (N < 50 samples) tests. Depending upon the results from the normality tests, statistical significance was analyzed with the *t*-test or the Mann–Whitney test for the comparison of normally distributed or nonparametric data, respectively. Statistical tests were significant if the *p*-value was < 0.05.

### 4.8. Metabolomic Characterization of the ΔBcstc3 and Overexpressed OvBcstc3 Mutant Strains

For the metabolomic characterization, the fungal strains were grown on malt agar medium (20 g/L D-glucose, 20 g/L malt extract, 1 g/L peptone, 20 g/L agar, pH 6.5–7) at 25 °C to produce mycelium plugs (0.8 mm) that were further used to inoculate medium for metabolite production. All studied strains were fermented in 40 Roux culture bottles (1000 mL), each containing 150 mL of modified Czapek-Dox medium (50 g glucose, 1 g yeast extract, 1 g K_2_HPO_4_, 2.5 g NaNO_3_, 0.5 g MgSO_4_⋅7H_2_O, 0.01 g FeSO_4_⋅7H_2_O, 0.005 g CuSO_4_⋅5H_2_O, pH 6.5–7, 1L of water). Bottles were inoculated with six mycelium plugs per bottle and incubated for 27 days at 25 °C on surface culture under daylight.

Then, 6 L of culture medium from each of the mutant strains was filtered under vacuum to remove mycelium, and the filtrates were saturated with NaCl and subjected to liquid−liquid extraction with ethyl acetate (EtOAc × 3) and dried over with anhydrous sodium sulphate. After filtration, the solvents were evaporated at reduced pressure to yield the crude extracts as yellow oils, 173 mg from (Δ*Bcstc3*) and 153 mg from (Ov*Bcstc3*). For metabolite isolation and characterization, the crude extracts were initially fractionated by column chromatography on silica gel, with a mixture of ethyl acetate/hexane containing increasing percentages (10−100%) of ethyl acetate. Different fractions were further purified by HPLC with a mixture of acetone, ethyl acetate and hexane. The metabolites purified were subjected to extensive spectroscopic analysis by ^1^H-NMR and ^13^C-NMR using 1D and 2D NMR, HRMS and IR techniques and their optical activity measured (α) for identification purposes.

## 5. Conclusions

The BcStc3 protein contains the conserved motifs in class I terpene cyclase involved in magnesium and substrate binding, essential for catalysis, as well as residues and structural domains related to the selectivity of the enzyme.

BcStc3 is a well-conserved protein among *Botrytis* species, especially in those that infect monocots, and are also in Basidiomycota and Ascomycota fungi but not in subphyla Taphrinomyctina and Saccharomycotina.

The *Bcstc3* gene is expressed in non-germinated conidia and is upregulated in planta and during vegetative growth. The relative abundance of *Bcstc3* transcripts affects the expression of the remaining *Bcstc*-encoding genes, suggesting differential crosstalk in the gene expression of the *Bcstc* gene family not only in conidia but also during vegetative growth and plant interaction.

The BcStc3 protein is involved in mycelium development and the secondary metabolites generated as its end products may affect the ability of *B. cinerea* to respond to osmotic stress and regulate ROS detoxification systems but not its production. The BcStc3 protein does not seem to be related to the production of conidia but plays a role in conidial morphogenesis and germination. The increased infection cushion found in the Ov*Bcstc3* strain is more likely related to the enhanced ROS generation induced by the BcStc3 overproduction than a direct role of the protein.

The *Bcstc3* deletion may modify the oxidative balance at the infection site, increasing the fungal capability to penetrate and invade host plant tissues.

## Figures and Tables

**Table 2:** Distribution of homologous proteins to BcStc3 in the kingdom Fungi.

Phylum	Sub-Phylum	Super-Class	Fungal Class	Number of Proteins	Number of Organisms
Ascomycota	Pezizomycotina	Leotiomyceta	Candelariomycetes	1	1
			Lecanoromycetes	9	9
			Eurotiomycetes	57	40
			Xylobotryomycetes	1	1
			Dothideomycetes	40	27
		Sordariomyceta	Leotiomycetes	11	11
			Sordariomycetes	34	32
Basidiomycota	Agaricomycotina		Agaricomycetes	7	3

## Data Availability

Data are contained within the article.

## Supplementary Materials

The following supporting information can be downloaded at: https://www.mdpi.com/article/10.3390/ijms25105125/s1.

## References

[1] Minami A., Ozaki T., Liu C., Oikawa H. (2018). Cyclopentane-forming di/sesterterpene synthases: Widely distributed enzymes in bacteria, fungi, and plants. Nat. Prod. Rep..

[2] Christianson D.W. (2008). Unearthing the roots of the terpenome. Curr. Opin. Chem. Biol..

[3] Tian S.-H., Zhang C., Zeng K.-W., Zhao M.-B., Jiang Y., Tu P.-F. (2018). Sesquiterpenoids from *Artemisia vestita*. Phytochemistry.

[4] Zhang C., Wen R., Ma X.-L., Zeng K.-W., Xue Y., Zhang P.-M., Zhao M.-B., Jiang Y., Liu G.-Q., Tu P.-F. (2018). Nitric oxide inhibitory sesquiterpenoids and its dimers from *Artemisia freyniana*. J. Nat. Prod..

[5] Xue G.-M., Li X.-Q., Chen C., Chen K., Wang X.-B., Gu Y.-C., Luo J.-G., Kong L.-Y. (2018). Highly oxidized guaianolide sesquiterpenoids with potential anti-inflammatory activity from *Chrysanthemum indicum*. J. Nat. Prod..

[6] Tan Y., Yang B., Lin X., Luo X., Pang X., Tang L., Liu Y., Li X., Zhou X. (2018). Nitrobenzoyl sesquiterpenoids with cytotoxic activities from a marine-derived *Aspergillus ochraceus* fungus. J. Nat. Prod..

[7] Kimani N.M., Matasyoh J.C., Kaiser M., Brun R., Schmidt T.J. (2018). Antiprotozoal sesquiterpene lactones and other constituents from *Tarchonanthus camphoratus* and *Schkuhria pinnata*. J. Nat. Prod..

[8] Wang W., Liu Y., Shi C., Pan L., Zhang X., Zou J.-J. (2022). High energy density renewable fuels based on multicyclic sesquiterpene: Synthesis and performance. Fuel.

[9] Whitehead J.N., Leferink N.G.H., Johannissen L.O., Hay S., Scrutton N.S. (2023). Decoding catalysis by terpene synthases. ACS Catal..

[10] T R., Sharma D., Lin F., Choong Y.K., Lim C., Jobichen C., Zhang C. (2023). Structural understanding of fungal terpene synthases for the formation of linear or cyclic terpene products. ACS Catal..

[11] González-Hernández R.A., Valdez-Cruz N.A., Macías-Rubalcava M.L., Trujillo-Roldán M.A. (2023). Overview of fungal terpene synthases and their regulation. World J. Microbiol. Biotechnol..

[12] Christianson D.W. (2006). Structural biology and chemistry of the terpenoid cyclases. Chem. Rev..

[13] Vavitsas K., Fabris M., Vickers C. (2018). Terpenoid metabolic engineering in photosynthetic microorganisms. Genes.

[14] Avalos M., Garbeva P., Vader L., van Wezel G.P., Dickschat J.S., Ulanova D. (2022). Biosynthesis, evolution and ecology of microbial terpenoids. Nat. Prod. Rep..

[15] Dai Q., Zhang F.-L., Feng T. (2021). Sesquiterpenoids specially produced by fungi: Structures, biological activities, chemical and biosynthesis (2015–2020). J. Fungi.

[16] Xu D., Xue M., Shen Z., Jia X., Hou X., Lai D., Zhou L. (2021). Phytotoxic secondary metabolites from fungi. Toxins.

[17] Shimada A., Kusano M., Takeuchi S., Fujioka S., Inokuchi T., Kimura Y. (2002). Aspterric acid and 6-Hydroxymellein, inhibitors of pollen development in *Arabidopsis thaliana*, produced by *Aspergillus terreus*. Z. Naturforsch. C.

[18] Dean R., van Kan J.A.L., Pretorius Z.A., Hammond-Kosack K.E., Di Pietro A., Spanu P.D., Rudd J.J., Dickman M., Kahmann R., Ellis J. (2012). The Top 10 fungal pathogens in molecular plant pathology. Mol. Plant Pathol..

[19] Dalmais B., Schumacher J., Moraga J., Le Pêcheur P., Tudzynski B., Collado I.G., Viaud M. (2011). The *Botrytis cinerea* phytotoxin botcinic acid requires two polyketide synthases for production and has a redundant role in virulence with botrydial. Mol. Plant Pathol..

[20] Colmenares A.J., Aleu J., Durán-Patrón R., Collado I.G., Hernández-Galán R. (2002). The putative role of botrydial and related metabolites in the infection mechanism of *Botrytis cinerea*. J. Chem. Ecol..

[21] da Silva Ripardo-Filho H., Coca Ruíz V., Suárez I., Moraga J., Aleu J., Collado I.G. (2023). From genes to molecules, secondary metabolism in *Botrytis cinerea*: New insights into anamorphic and teleomorphic stages. Plants.

[22] Rossi F.R., Gárriz A., Marina M., Romero F.M., Gonzalez M.E., Collado I.G., Pieckenstain F.L., Rubén Rossi F., Gárriz A., Marina M. (2011). The sesquiterpene botrydial produced by *Botrytis cinerea* induces the hypersensitive response on plant tissues and its action is modulated by salicylic acid and jasmonic acid signaling. Mol. Plant-Microbe Interact..

[23] D’Ambrosio J.M., Gonorazky G., Sueldo D.J., Moraga J., Di Palma A.A., Lamattina L., Collado I.G., Laxalt A.M. (2018). The sesquiterpene botrydial from *Botrytis cinerea* induces phosphatidic acid production in tomato cell suspensions. Planta.

[24] Malmierca M.G., Izquierdo-Bueno I., Mccormick S.P., Cardoza R.E., Alexander N.J., Moraga J., Gomes E.V., Proctor R.H., Collado I.G., Monte E. (2016). Botrydial and botcinins produced by *Botrytis cinerea* regulate the expression of *Trichoderma arundinaceum* genes involved in trichothecene biosynthesis. Mol. Plant Pathol..

[25] Vignatti P., Gonzalez M.E., Jofré E.C., Bolívar-Anillo H.J., Moraga J., Viaud M., Collado I.G., Pieckenstain F.L. (2020). Botrydial confers *Botrytis cinerea* the ability to antagonize soil and phyllospheric bacteria. Fungal Biol..

[26] Amselem J., Cuomo C.A., van Kan J.A.L.L., Viaud M., Benito E.P., Couloux A., Coutinho P.M., de Vries R.P., Dyer P.S., Fillinger S. (2011). Genomic analysis of the necrotrophic fungal pathogens *Sclerotinia sclerotiorum* and *Botrytis cinerea*. PLoS Genet..

[27] Porquier A., Morgant G., Moraga J., Dalmais B., Luyten I., Simon A., Pradier J.-M., Amselem J., Collado I.G., Viaud M. (2016). The botrydial biosynthetic gene cluster of *Botrytis cinerea* displays a bipartite genomic structure and is positively regulated by the putative Zn(II)2Cys6 transcription factor BcBot6. Fungal Genet. Biol..

[28] Pinedo C., Wang C.M., Pradier J.M., Dalmais B., Choquer M., Le Pêcheur P., Morgant G., Collado I.G., Cane D.E., Viaud M. (2008). Sesquiterpene synthase from the botrydial biosynthetic gene cluster of the phytopathogen *Botrytis cinerea*. ACS Chem. Biol..

[29] Izquierdo-Bueno I., González-Rodríguez V.E., Simon A., Dalmais B., Pradier J., Le Pêcheur P., Mercier A., Walker A., Garrido C., Collado I.G. (2018). Biosynthesis of abscisic acid in fungi: Identification of a sesquiterpene cyclase as the key enzyme in *Botrytis cinerea*. Environ. Microbiol..

[30] Suárez I., González-Rodríguez V.E., Viaud M., Garrido C., Collado I.G. (2020). Identification of the sesquiterpene cyclase involved in the biosynthesis of (+)-4-Epi-eremophil-9-en-11-ol Derivatives isolated from *Botrytis cinerea*. ACS Chem. Biol..

[31] Suárez I., da Silva Lima G., Conti R., Pinedo C., Moraga J., Barúa J., de Oliveira A.L.L., Aleu J., Durán-Patrón R., Macías-Sánchez A.J. (2018). Structural and biosynthetic studies on eremophilenols related to the phytoalexin capsidiol, produced by *Botrytis cinerea*. Phytochemistry.

[32] Suárez I., Pinedo C., Aleu J., Durán-Patrón R., Macías-Sánchez A.J., Hernández-Galán R., Collado I.G. (2022). The complemented mutant Δ*Bcstc7*, in the STC7 of *Botrytis cinerea* led to the characterization of 11,12,13-tri-nor-eremophilenols derivatives. Phytochemistry.

[33] Pinto A.A., Barúa J.E., Almeida M.O., Viaud M., Zorrilla D., Collado I.G., Macías-Sánchez A.J., Durán-Patrón R. (2022). Structural and biosynthetic studies of botrycinereic acid, a new cryptic metabolite from the fungus *Botrytis cinerea*. Bioorg. Chem..

[34] Paysan-Lafosse T., Blum M., Chuguransky S., Grego T., Pinto B.L., Salazar G.A., Bileschi M.L., Bork P., Bridge A., Colwell L. (2023). InterPro in 2022. Nucleic Acids Res..

[35] Marchler-Bauer A., Derbyshire M.K., Gonzales N.R., Lu S., Chitsaz F., Geer L.Y., Geer R.C., He J., Gwadz M., Hurwitz D.I. (2015). CDD: NCBI’s conserved domain database. Nucleic Acids Res..

[36] Lu S., Wang J., Chitsaz F., Derbyshire M.K., Geer R.C., Gonzales N.R., Gwadz M., Hurwitz D.I., Marchler G.H., Song J.S. (2020). CDD/SPARCLE: The conserved domain database in 2020. Nucleic Acids Res..

[37] Chang H., Cheng T., Wang A.H.-J. (2021). Structure, catalysis, and inhibition mechanism of prenyltransferase. IUBMB Life.

[38] Aaron J.A., Christianson D.W. (2010). Trinuclear metal clusters in catalysis by terpenoid synthases. Pure Appl. Chem..

[39] Nosenko T., Zimmer I., Ghirardo A., Köllner T.G., Weber B., Polle A., Rosenkranz M., Schnitzler J.-P. (2023). Predicting functions of putative fungal sesquiterpene synthase genes based on multiomics data analysis. Fungal Genet. Biol..

[40] Lou T., Li A., Xu H., Pan J., Xing B., Wu R., Dickschat J.S., Yang D., Ma M. (2023). Structural insights into three sesquiterpene synthases for the biosynthesis of tricyclic sesquiterpenes and chemical space expansion by structure-based mutagenesis. J. Am. Chem. Soc..

[41] Varadi M., Anyango S., Deshpande M., Nair S., Natassia C., Yordanova G., Yuan D., Stroe O., Wood G., Laydon A. (2022). AlphaFold protein structure database: Massively expanding the structural coverage of protein-sequence space with high-accuracy models. Nucleic Acids Res..

[42] López-Gallego F., Wawrzyn G., Schmidt-Dannert C. (2010). Selectivity of fungal sesquiterpene synthases: Role of the active site’s H-1α loop in catalysis. Appl. Environ. Microbiol..

[43] Marchler-Bauer A., Bo Y., Han L., He J., Lanczycki C.J., Lu S., Chitsaz F., Derbyshire M.K., Geer R.C., Gonzales N.R. (2017). CDD/SPARCLE: Functional classification of proteins via subfamily domain architectures. Nucleic Acids Res..

[44] Kanehisa M., Sato Y., Morishima K. (2016). BlastKOALA and GhostKOALA: KEGG tools for functional characterization of genome and metagenome sequences. J. Mol. Biol..

[45] Pearson W.R. (2013). An Introduction to sequence similarity (“Homology”) searching. Curr. Protoc. Bioinforma..

[46] Choquer M., Rascle C., Gonçalves I.R., de Vallée A., Ribot C., Loisel E., Smilevski P., Ferria J., Savadogo M., Souibgui E. (2021). The infection cushion of *Botrytis cinerea*: A fungal ‘weapon’ of plant-biomass destruction. Environ. Microbiol..

[47] Tani H., Koshino H., Sakuno E., Cutler H.G., Nakajima H. (2006). Botcinins E and F and botcinolide from *Botrytis cinerea* and structural revision of botcinolides. J. Nat. Prod..

[48] Pinedo C., Moraga J., Barua J., González-Rodríguez V.E., Aleu J., Durán-Patrón R., Macías-Sánchez A.J., Hanson J.R., Viaud M., Hernández-Galán R. (2016). Chemically induced cryptic sesquiterpenoids and expression of sesquiterpene cyclases in botrytis cinerea revealed new sporogenic (+)-4-epi-eremophil-9-en-11-ols. ACS Chem. Biol..

[49] Nikolaiczyk V., Irwan J., Nguyen T., Fohrer J., Elbers P., Schrank P., Davari M.D., Kirschning A. (2023). Rational reprogramming of the sesquiterpene synthase BcBOT2 yields new terpenes with presilphiperfolane skeleton. Catal. Sci. Technol..

[50] Valero-Jiménez C.A., Veloso J., Staats M., van Kan J.A.L. (2019). Comparative genomics of plant pathogenic *Botrytis* species with distinct host specificity. BMC Genom..

[51] Valero-Jiménez C.A., Steentjes M.B.F., Slot J.C., Shi-Kunne X., Scholten O.E., van Kan J.A.L. (2020). Dynamics in secondary metabolite gene clusters in otherwise highly syntenic and stable genomes in the fungal genus *Botrytis*. Genome Biol. Evol..

[52] Garfinkel A.R. (2021). The History of *Botrytis* taxonomy, the rise of phylogenetics, and implications for species recognition. Phytopathology®.

[53] Hage H., Couillaud J., Salamov A., Loussouarn-Yvon M., Durbesson F., Ormeño E., Grisel S., Duquesne K., Vincentelli R., Grigoriev I. (2023). An HMM approach expands the landscape of sesquiterpene cyclases across the kingdom fungi. Microb. Genom..

[54] Robey M.T., Caesar L.K., Drott M.T., Keller N.P., Kelleher N.L. (2021). An interpreted atlas of biosynthetic gene clusters from 1,000 fungal genomes. Proc. Natl. Acad. Sci. USA.

[55] He H., Bian G., Herbst-Gervasoni C.J., Mori T., Shinsky S.A., Hou A., Mu X., Huang M., Cheng S., Deng Z. (2020). Discovery of the cryptic function of terpene cyclases as aromatic prenyltransferases. Nat. Commun..

[56] Masi M., Meyer S., Górecki M., Pescitelli G., Clement S., Cimmino A., Evidente A. (2018). Phytotoxic Activity of Metabolites isolated from *Rutstroemia* sp.n., the causal agent of bleach blonde syndrome on cheatgrass (*Bromus tectorum*). Molecules.

[57] Díaz-Escandón D., Tagirdzhanova G., Vanderpool D., Allen C.C.G., Aptroot A., Češka O., Hawksworth D.L., Huereca A., Knudsen K., Kocourková J. (2022). Genome-level analyses resolve an ancient lineage of symbiotic ascomycetes. Curr. Biol..

[58] Voglmayr H., Fournier J., Jaklitsch W.M. (2019). Two new classes of Ascomycota: Xylobotryomycetes and Candelariomycetes. Persoonia-Mol. Phylogeny Evol. Fungi.

[59] Schafhauser T., Wibberg D., Binder A., Rückert C., Busche T., Wohlleben W., Kalinowski J. (2022). Genome assembly and genetic traits of the pleuromutilin-producer *Clitopilus passeckerianus* DSM1602. J. Fungi.

[60] Tesei D. (2022). Black fungi research: Out-of-this-world implications. Encyclopedia.

[61] Wu J., Yang X., Duan Y., Wang P., Qi J., Gao J.-M., Liu C. (2022). Biosynthesis of sesquiterpenes in Basidiomycetes: A Review. J. Fungi.

[62] Kendrick B. (2003). Ainsworth bisbys dictionary of the fungi a review. Mycologist.

[63] Dörner S., Rogge K., Fricke J., Schäfer T., Wurlitzer J.M., Gressler M., Pham D.N.K., Manke D.R., Chadeayne A.R., Hoffmeister D. (2022). Genetic survey of psilocybe natural products. ChemBioChem.

[64] Zhang C., Chen X., Orban A., Shukal S., Birk F., Too H.-P., Rühl M. (2020). *Agrocybe aegerita* serves as a gateway for identifying sesquiterpene biosynthetic enzymes in higher fungi. ACS Chem. Biol..

[65] de Sena Filho J.G., Quin M.B., Spakowicz D.J., Shaw J.J., Kucera K., Dunican B., Strobel S.A., Schmidt-Dannert C. (2016). Genome of Diaporthe sp. provides insights into the potential inter-phylum transfer of a fungal sesquiterpenoid biosynthetic pathway. Fungal Biol..

[66] Spatafora J.W., Bushley K.E. (2015). Phylogenomics and evolution of secondary metabolism in plant-associated fungi. Curr. Opin. Plant Biol..

[67] Schäfer E., Seibold P.S., Bartram S., Trottmann F., Haensch V.G., Gressler M., Chadeayne A.R., Hertweck C., O’Connor S.E., Hoffmeister D. (2023). A “Magic mushroom” multi-product sesquiterpene synthase. ChemBioChem.

[68] Montibus M., Pinson-Gadais L., Richard-Forget F., Barreau C., Ponts N. (2015). Coupling of transcriptional response to oxidative stress and secondary metabolism regulation in filamentous fungi. Crit. Rev. Microbiol..

[69] Umar A., Darwish D.B.E., Albalwe F.M. (2024). Fungal secondary metabolites and their role in stress management. Fungal Secondary Metabolites.

[70] Overy D., Correa H., Roullier C., Chi W.-C., Pang K.-L., Rateb M., Ebel R., Shang Z., Capon R., Bills G. (2017). Does osmotic stress affect natural product expression in Fungi?. Mar. Drugs.

[71] Ochiai N., Tokai T., Nishiuchi T., Takahashi-Ando N., Fujimura M., Kimura M. (2007). Involvement of the osmosensor histidine kinase and osmotic stress-activated protein kinases in the regulation of secondary metabolism in *Fusarium graminearum*. Biochem. Biophys. Res. Commun..

[72] Fountain J.C., Bajaj P., Pandey M., Nayak S.N., Yang L., Kumar V., Jayale A.S., Chitikineni A., Zhuang W., Scully B.T. (2016). Oxidative stress and carbon metabolism influence *Aspergillus flavus* transcriptome composition and secondary metabolite production. Sci. Rep..

[73] Zhang G., Zhang C., Leng D., Yan P., Wang Z., Zhang M., Wu Z. (2021). The non-canonical functions of telomerase reverse transcriptase gene GlTert on regulating fungal growth, oxidative stress, and ganoderic acid biosynthesis in *Ganoderma lucidum*. Appl. Microbiol. Biotechnol..

[74] Wang Z., Lopez-Giraldez F., Slot J., Yarden O., Trail F., Townsend J.P. (2022). Secondary metabolism gene clusters exhibit increasingly dynamic and differential expression during asexual growth, conidiation, and sexual development in *Neurospora crassa*. mSystems.

[75] Wang Y., Wu J., Yan J., Guo M., Xu L., Hou L., Zou Q. (2022). Comparative genome analysis of plant ascomycete fungal pathogens with different lifestyles reveals distinctive virulence strategies. BMC Genom..

[76] Udompaisarn S., Toopaang W., Sae-Ueng U., Srisuksam C., Wichienchote N., Wasuwan R., Nahar N.A.S., Tanticharoen M., Amnuaykanjanasin A. (2020). The polyketide synthase PKS15 has a crucial role in cell wall formation in *Beauveria bassiana*. Sci. Rep..

[77] Choquer M., Fournier E., Kunz C., Levis C., Pradier J.-M., Simon A., Viaud M. (2007). *Botrytis cinerea* virulence factors: New insights into a necrotrophic and polyphageous pathogen. FEMS Microbiol. Lett..

[78] de Vallée A., Bally P., Bruel C., Chandat L., Choquer M., Dieryckx C., Dupuy J.W., Kaiser S., Latorse M.-P., Loisel E. (2019). A similar secretome disturbance as a hallmark of non-pathogenic *Botrytis cinerea* ATMT-mutants?. Front. Microbiol..

[79] Zhang C., He Y., Zhu P., Chen L., Wang Y., Ni B., Xu L. (2015). Loss of *bcbrn1* and *bcpks13* in *Botrytis cinerea* not only blocks melanization but also increases vegetative growth and virulence. Mol. Plant-Microbe Interact..

[80] Todd J.N.A., Carreón-Anguiano K.G., Islas-Flores I., Canto-Canché B. (2022). Fungal effectoromics: A world in constant evolution. Int. J. Mol. Sci..

[81] Collemare J., O’Connell R., Lebrun M. (2019). Nonproteinaceous effectors: The terra incognita of plant–fungal interactions. New Phytol..

[82] Rangel L.I., Bolton M.D. (2022). The unsung roles of microbial secondary metabolite effectors in the plant disease cacophony. Curr. Opin. Plant Biol..

[83] Camacho C., Coulouris G., Avagyan V., Ma N., Papadopoulos J., Bealer K., Madden T.L. (2009). BLAST+: Architecture and applications. BMC Bioinform..

[84] Robert X., Gouet P. (2014). Deciphering key features in protein structures with the new ENDscript server. Nucleic Acids Res..

[85] Tamura K., Stecher G., Kumar S. (2021). MEGA11: Molecular evolutionary genetics analysis version 11. Mol. Biol. Evol..

[86] Jumper J., Evans R., Pritzel A., Green T., Figurnov M., Ronneberger O., Tunyasuvunakool K., Bates R., Žídek A., Potapenko A. (2021). Highly accurate protein structure prediction with AlphaFold. Nature.

[87] González-Rodríguez V.E., Garrido C., Cantoral J.M., Schumacher J. (2016). The F-actin capping protein is required for hyphal growth and full virulence but is dispensable for septum formation in *Botrytis cinerea*. Fungal Biol..

[88] Mansfield J.W. (1985). Fungal nutrition and physiology. Physiol. Plant Pathol..

[89] van der Vlugt-Bergmans C.J.B., Wagemakers C.A.M., van Kan J.A.L. (1997). Cloning and Expression of the cutinase A gene of *Botrytis cinerea*. Mol. Plant-Microbe Interact..

[90] Schumacher J. (2012). Tools for *Botrytis cinerea*: New expression vectors make the gray mold fungus more accessible to cell biology approaches. Fungal Genet. Biol..

[91] Colot H.V., Park G., Turner G.E., Ringelberg C., Crew C.M., Litvinkova L., Weiss R.L., Borkovich K.A., Dunlap J.C. (2006). A high-throughput gene knockout procedure for *Neurospora* reveals functions for multiple transcription factors. Proc. Natl. Acad. Sci. USA.

[92] Livak K.J., Schmittgen T.D. (2001). Analysis of relative gene expression data using real-time quantitative PCR and the 2^−ΔΔCT^ method. Methods.

[93] Cui K., He L., Zhao Y., Mu W., Lin J., Liu F. (2021). Comparative analysis of *Botrytis cinerea* in response to the microbial secondary metabolite benzothiazole using iTRAQ-Based quantitative proteomics. Phytopathology®.

[94] Schneider C.A., Rasband W.S., Eliceiri K.W. (2012). NIH Image to ImageJ: 25 years of image analysis. Nat. Methods.

[95] Sekulska-Nalewajko J., Gocławski J., Chojak-Koźniewska J., Kuźniak E. (2016). Automated image analysis for quantification of reactive oxygen species in plant leaves. Methods.

