# Supplementary material for: Unravelling the Function of the Sesquiterpene Cyclase STC3 in the Lifecycle of Botrytis cinerea
Source: Int J Mol Sci. 2024 May 8;25(10):5125. doi: 10.3390/ijms25105125 (PMC11120764; doi:10.3390/ijms25105125)
Supplement: Supplementary file 1 [file ijms-25-05125-s001.zip › Supporting information_2_ver.pdf]

Figure S1. (A) The conservation pattern of the BcStc3 sequence calculated by Con Surf server. Amino acid residues are colored turquoise to bordeaux according to the conservation scores. Green boxes indicate the two conserved magnesium binding motifs. Orange box denotes conserved effector triad. Blue box indicates conserved R and Y residues involved in substrate binding. Red box indicates H1α-loop ending at the conserved asparagine (N329) residue**. (B) Position-specific evolutionary conservation scores of each residue of BcStc3.** Scores were calculated with AL2CO (http://prodata.swmed.edu/al2co) for an alignment of 160 BcStc3-like proteins downloaded from NCBI and aligned with Clustal Omega (https://www.ebi.ac.uk/services). Conservation indexes were averaged in windows of seven residues to smooth the graph. The conserved residues of the canonical motifs of the class I terpenoid cyclases are indicated and colored according to the boxes in (A).

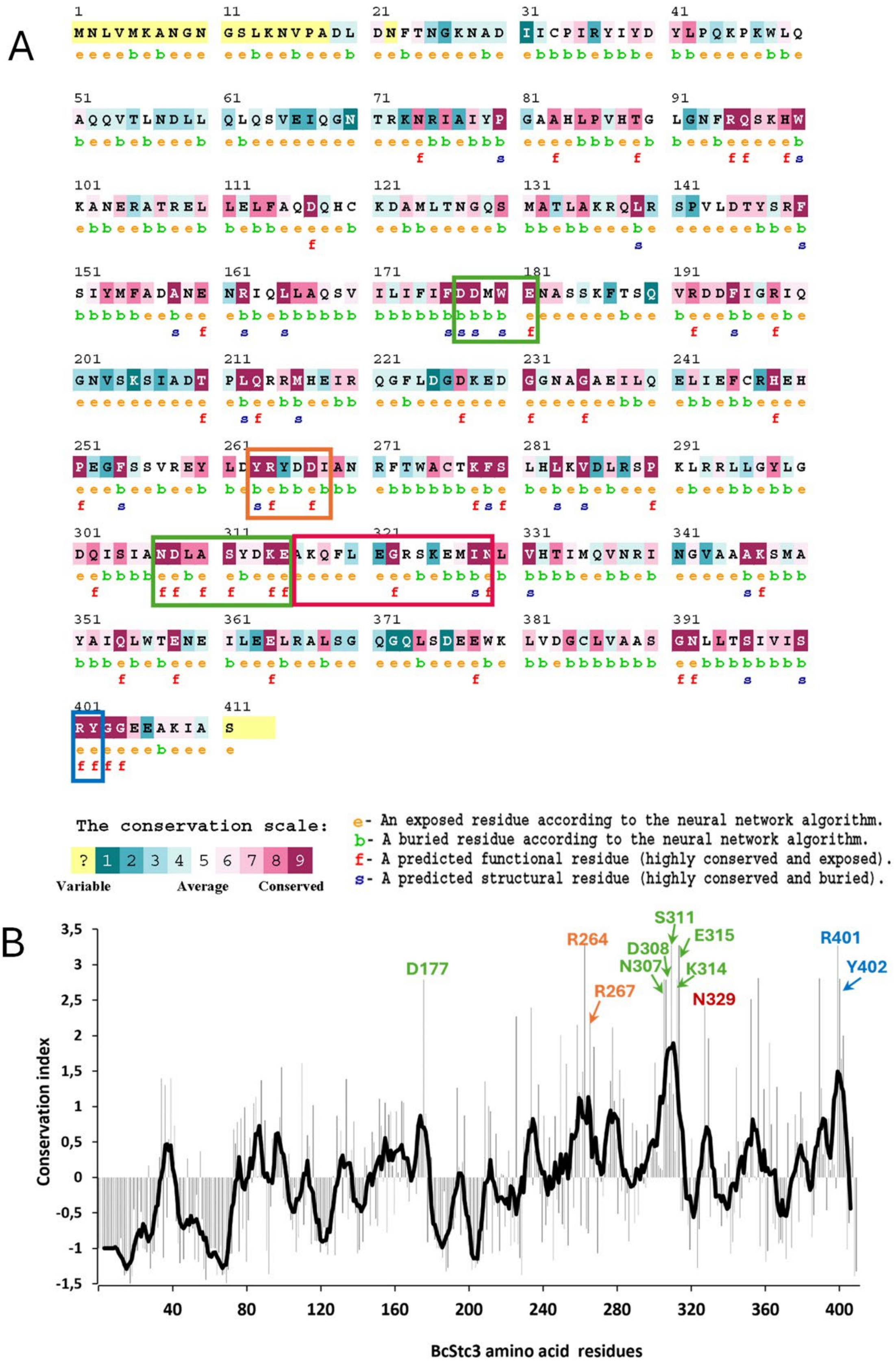
A
1 MNLVMKANGN 11 GSLKNVPADL 21 DNFTNGKNAD 31 IICPIRYIYD 41 YLPQKPKWLQ
51 AQQVTLNDLL 61 QLQSVEIQGN 71 TRKNRIAIYP 81 GAAHLPVHTG 91 LGNFRQSKHW
101 KANERATREL 111 LELFAQDQHC 121 KDAMLTNGQS 131 MATLAKRQLR 141 SPVLDTYSRF
151 SIYMFADANE 161 NRIQLLAQSV 171 ILIFIFDDMW 181 ENASSKFTSQ 191 VRDDFIGRIQ
201 GNVSKSIADT 211 PLQRRMHEIR 221 QGFLDGDKED 231 GGNAGAEILQ 241 ELIEFCRHEH
251 PEGFSSVREY 261 LDYRYDDIAN 271 RFTWACTKFS 281 LHLKVDLRSP 291 KLRRLLGYLG
301 DQISIANDLA 311 SYDKEAKQFL 321 EGRSKEMINL 331 VHTIMQVNRI 341 NGVAAAKSMA
351 YAIQLWTENE 361 ILEELRALSG 371 QGQLSDEEWK 381 LVDGCLVAAS 391 GNLLTSIVIS
401 RYGGEEAKIA 411 S
The conservation scale:
? 1 2 3 4 5 6 7 8 9
Variable
Average
Conserved
e- An exposed residue according to the neural network algorithm.
b- A buried residue according to the neural network algorithm.
f- A predicted functional residue (highly conserved and exposed).
s- A predicted structural residue (highly conserved and buried).
B
Conservation index
BcStc3 amino acid residues
D177
R264
R267
N307
D308
S311
E315
K314
N329
R401
Y402

Figure S2. Alignment of BcStc3 and homologous protein sequences of other *Botrytis* species. Protein sequences were selected after running similarity search by BlastP using BcStc3 as query sequence. Multiple alignments of protein sequences were carried out with Clustal-Omega and were visualized using ESPript 3.0. Green boxes indicate the two conserved magnesium binding motifs. Orange box denotes conserved effector triad. Blue box indicates conserved R and Y residues involved in substrate binding. Red box indicates H1α-loop ending at the conserved asparagine (N329) residue. Bcin, *Botrytis cinerea;* Btu, *Botrytis tulipae*; Bsin, *Botrytis sinoallii*; Bell, *Botrytis elliptica*; Bdew, *Botrytis deweyae*; Bgal, *Botrytis galanthina*; Bhya, *Botrytis hyacinthi*; Bbys, *Botrytis byssoidea*; Bglo, *Botryotinia globosa*; Bnar, *Botryotinia narcissicola.* NCBI accession number of each sequence is shown.

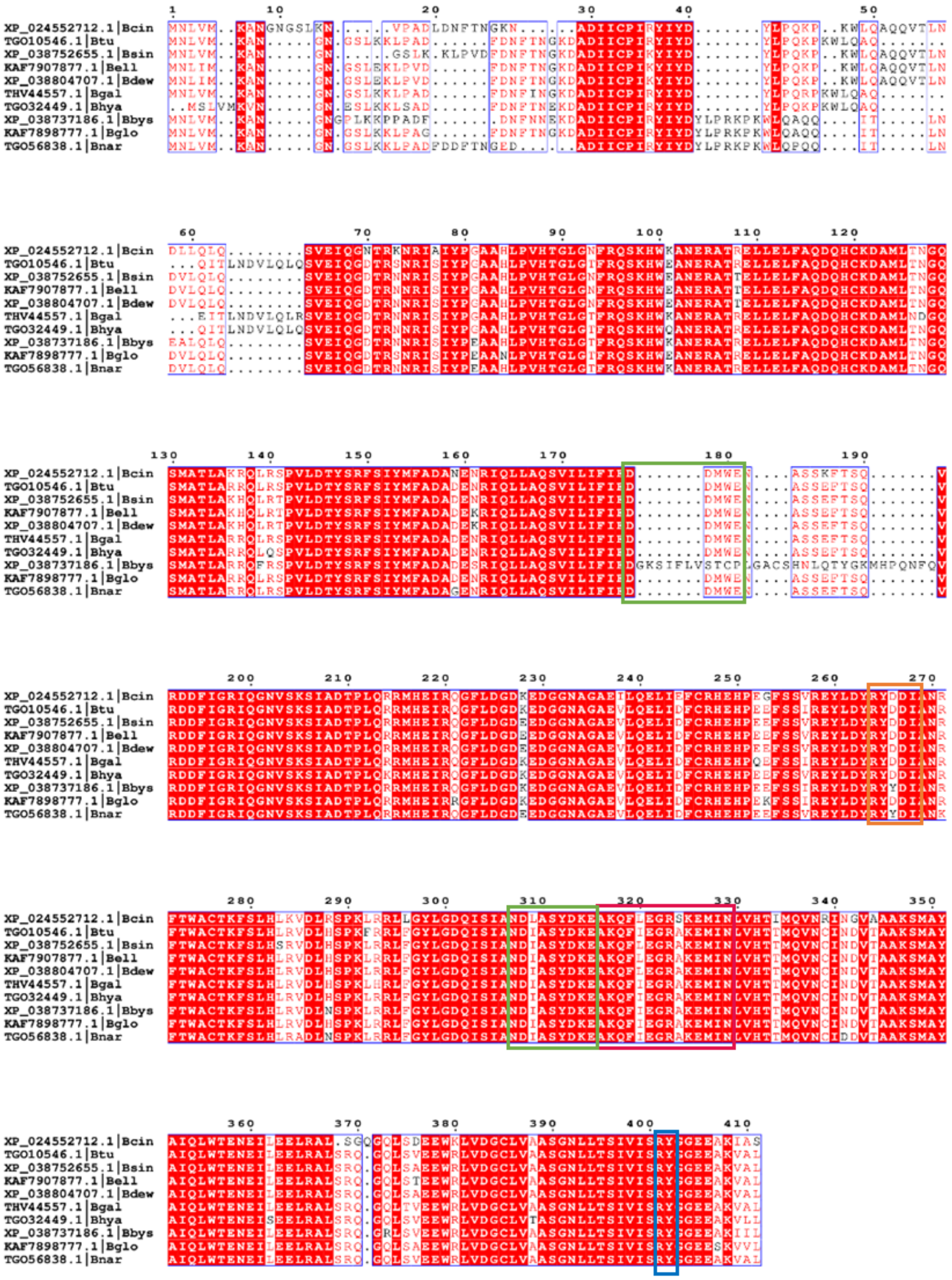

Figure S3. Alignment of BcStc3 and homologous protein sequences of other fungi species. It was selected at least one protein from each of the fungal taxonomic orders in which homologous proteins to BcStc3 had been identified among the first 55 sequences retrieved from the BlastP search using BcStc3 as the query sequence. Multiple alignments of protein sequences were carried out with Clustal-Omega and were visualized using ESPript 3.0. Green boxes indicate the two conserved magnesium binding motifs. Orange box denotes conserved effector triad. Blue box indicates conserved R and Y residues involved in substrate binding. Red box indicates H1α-loop ending at the conserved asparagine (N329) residue. Bot_cin, *Botrytis cinerea;* Rutst_sp, *Rutstroemia sp* (Leotiomycetes, Helotiales); Pyc_pra, *Pycnora praestabilis* (Candelariomycetes); Sei_lac, *Seirophora lacunosa* (Lecanoromycetes); Sti_urc, *Stictis urceolata* (Lecanoromycetes); Exo_mes, *Exophiala m4esophile* (Eurotiomycetes, Chaetothryales); Cir_nov, *Cirrosporium novae-zelandiae* (Xylobotryomycetes); Alt_pan, *Alternaria panax* (Dothideomycetes, Pleorales); Neo_par, *Neofusicoccum parvum* (Dothideomycetes, Botryosphaeriales); Dia_amy, *Diaporthe* amygdali (Sordariomycetes, Draprthales); Dot_sym,*Dothidotthia symphoricarpi* (Dothideomycetes, Pleorales); Coll_tam, *Colletotrichum tamarilloi* (Sordariomycetes, Glomerellales); Micr_niv, *Microdochium nivale* (Sordariomycetes, Xylariales); Gal_mar, *Galerina marginata* (Basidiomycota, Agaricomycetes). NCBI accession number of each sequence is shown.

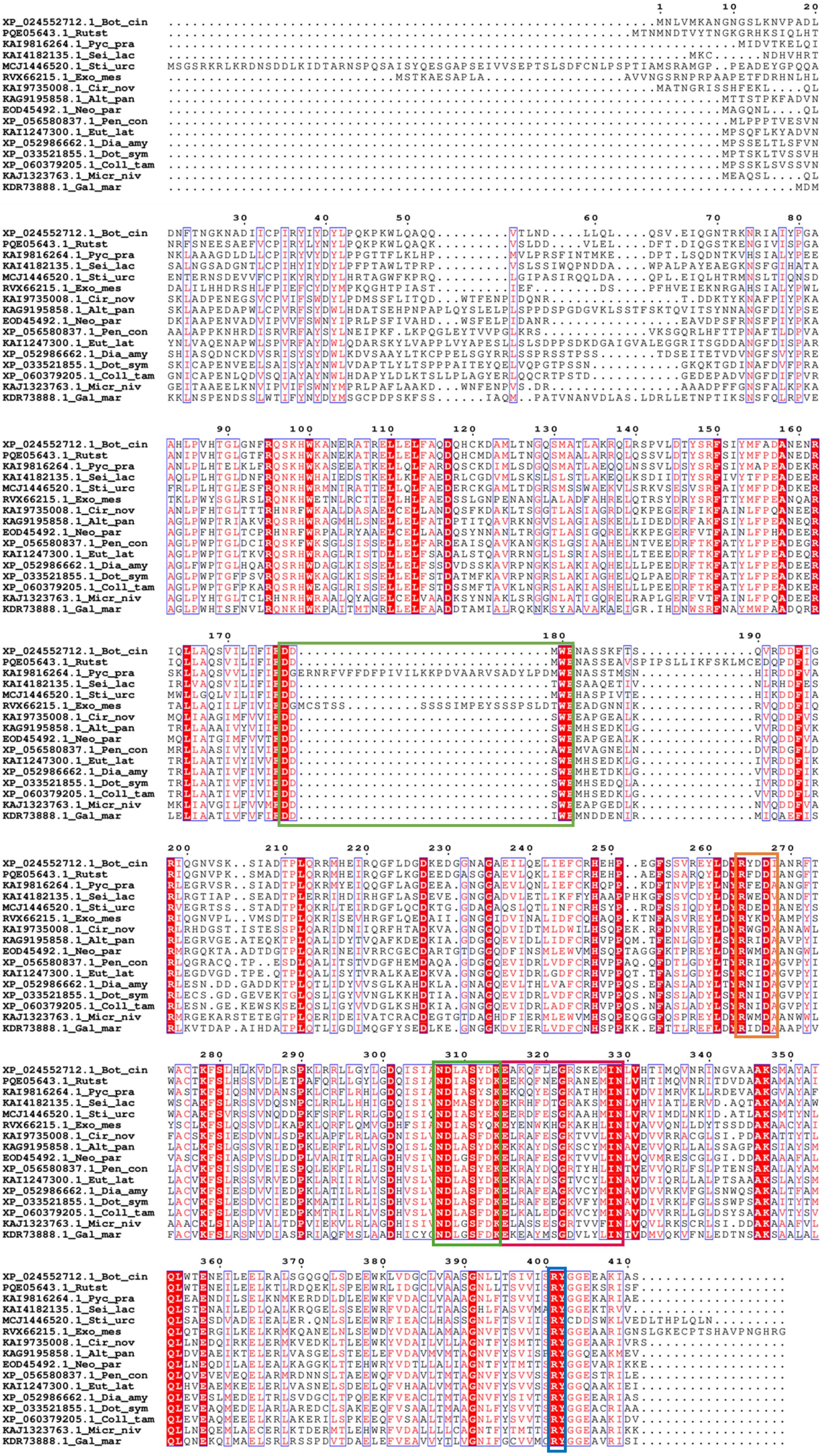
XP_024552712.1_Bot_cin
PQE05643.1_Rutst
KAI9816264.1_Pyc_pra
KAI4182135.1_Sei_lac
MCJ1446520.1_Sti_urc
RVX66215.1_Exo_mes
KAI9735008.1_Cir_nov
KAG9195858.1_Alt_pan
EOD45492.1_Neo_par
XP_056580837.1_Pen_con
KAI1247300.1_Eut_lat
XP_052986662.1_Dia_amy
XP_033521855.1_Dot_sym
XP_060379205.1_Coll_tam
KAJ1323763.1_Micr_niv
KDR73888.1_Gal_mar

Figure S4. **Gene expression of the *Bstc* gene family in the *B. cinerea* B05.10 strain. (A)** Relative expression levels in non-germinated conidia. mRNA levels were quantified by qRT-PCR, normalized to *actA* and *B-tub* mRNA levels, and expressed as a fold change over *Bcstc1*. **(B)** Relative expression levels in axenic culture and *in planta*. mRNA levels were quantified by qRT-PCR in mycelium grown in YGG medium (axenic culture) and in leaves of N. tabaccum infected with a conidial suspension (*in planta*). Values were normalized to *actA* and *B-tub*mRNA levels and expressed as a fold change over mRNA levels of each gene in non-germinated conidia. Error bars show standard deviation of three biological replicates (n = 3). Different letters above the bars in (A) mean significant differences between n fold changes of the six genes (*p*-value<0.05). * in (B) means significant differences between mRNA levels in axenic and in planta and axenic culture for each gene (*p*-value<0.05).

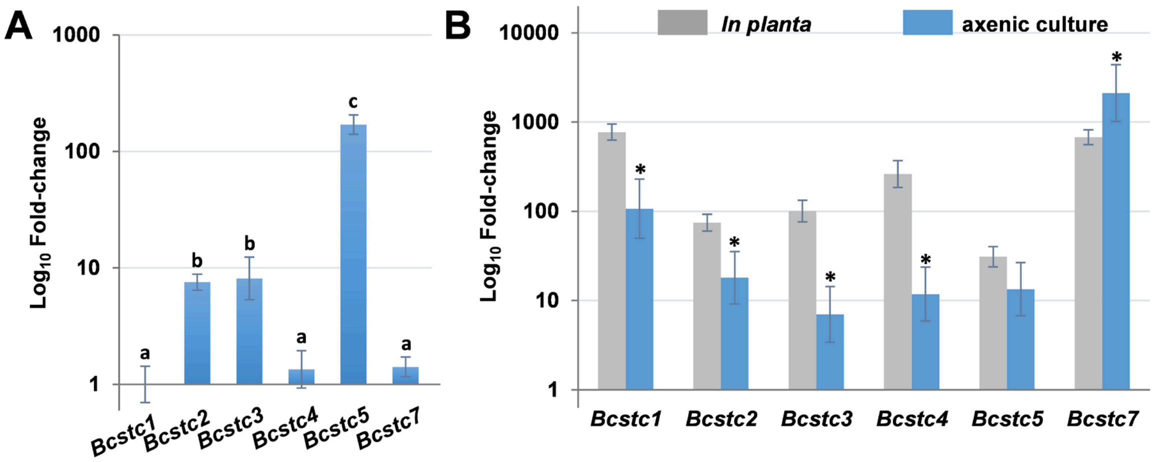

Figure S5. Virulence assays. Different hosts were inoculated with 5 µL droplets containing 2500 conidia of the indicated strain in TGGK solution, incubated in the dark at 20°C under conditions of high humidity and the degree of damage was estimated. **(A)** The damage on inoculated tomato fruits was estimated using a qualitative scale ranging from 1 (very good appearance) to 4 (bad appearance) and results are presented as the percentage of fruits in each disease grade to the total number of infected fruits at 2, 3 and 4 days post-inoculation. **(B)** A qualitative scale ranging from 1 (very good) to 4 (bad appearance) was used to estimate the damage on inoculated grapefruits and results are presented as the percentage of fruits in each disease grade to the total number of infected fruits at 2-, 3- and 4-days post-inoculation. **(C)** Representative images of the lesions produced by each fungal strain on gerbera and **(D)** detached tobacco leaves 4 days after inoculation.

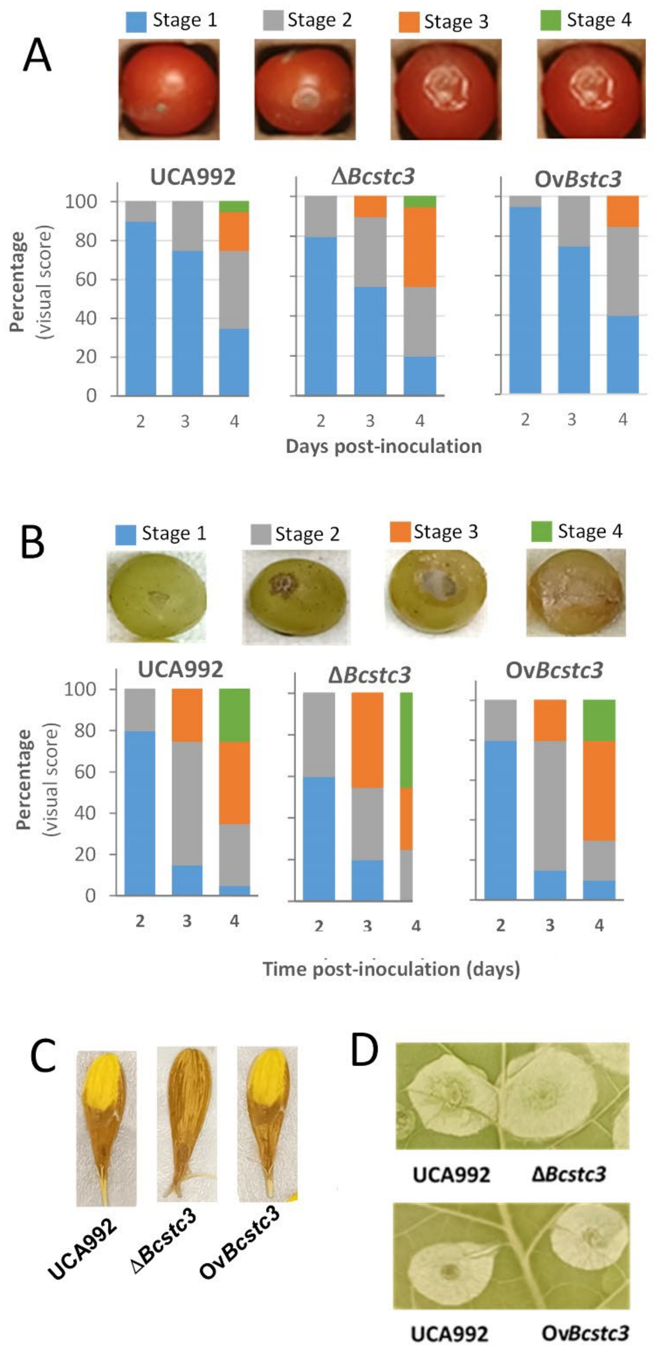

Figure S6. Secondary metabolites isolated from the fermentation of the Δ*Bcstc3* or Ov*Bcstc3* strains.

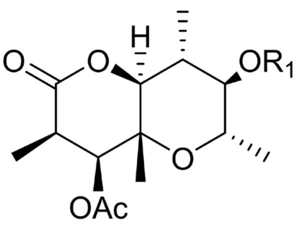

1 Botcinin A

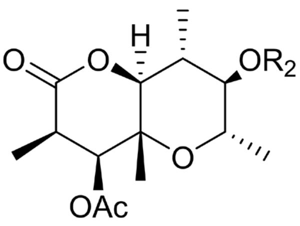

2 Botcinin B

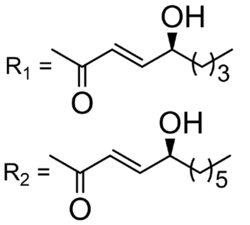

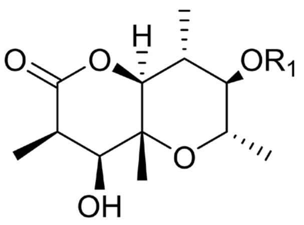

3 Botcinin E

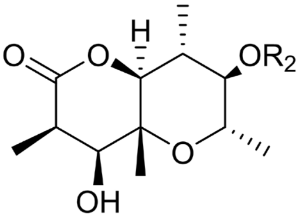

4 Homobotcinin E

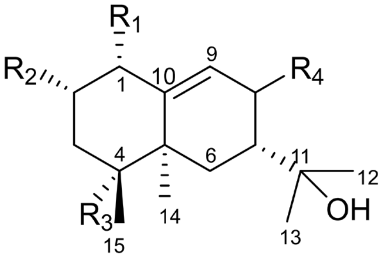

**5**	R_1 = OH	R_2= R_3= R_4= H
**6**	R_1 = O	R_2= R_3= R_4= H
**7**	R_1 = H	R_2= OH R_3= R_4= H
**8**	R_1 = H	R_2= H R_3= OH H R_4= O
**9**	R_1 = H	R_2= OH R_3= H R_4= αOH
**10**	R_1 = OH	R_2= OH R_3= R_4= H
**11**	R_1 = H	R_2= OH R_3= H R_4= βOH
**12**	R_1 = H	R_2= H R_3= OH R_4= βOH
**13**	R_1 = O	R_2= R_3= H R_4= βOH
**14**	R_1 = H	R_2= H R_3= OH R_4= H

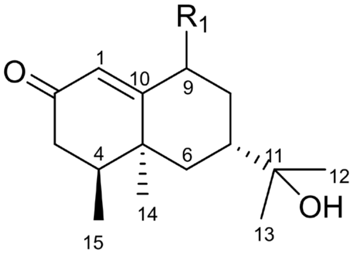

15 R_1 = O
16 R_1 = H

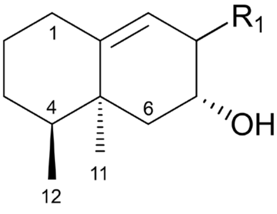

17 R_1 = βOH
18 R_1 = αOH

Table S1. Oligonucleotides used in this work.

Primer	Sequence (5' → 3')	Features	Used for	References
***Hph*-F**	GTCGGAGACAGAAGATGATATTGAAGGAGC	P*trpC*	Cloning – amplification of P*trpC*::*hph* for Δ*bcstc3* construct	[1]
***Hph*-R**	GTTGGAGATTTCAGTAACGTTAAGTGGAT	*Hph*	Cloning – amplification of P*trpC*::*hph* for Δ*bcstc3* construct	[1]
***TrpC*-P2**	CCTCCACTAGCTCCAGCCAAGCCC	P*trpC*	Diagnostic PCR – homologous integration at *bcstc3*-5'	[1]
***TrpC*-T**	GGAATAGAGTAGATGCCGACCGG	T*trpC*	Diagnostic PCR – homologous integration at *bcstc3*-3'	[1]
***Bcstc3*-5F**	GTAACGCCAGGGTTTTCCCAGTCACGACGTT TGTCTGCCCTTTGACGGA	pRS426-5F – *bcstc3*-5'	Cloning KO construct– amplification of 5' flank for Δ*bcstc3* construct	This study
***Bcstc3*-5R**	ATCCACTTAACGTTACTGAAATCTCCAA CCCAAGCCGTTAATGATTCG	*Hph* – *bcstc3*-5'	Cloning – amplification of 5' flank for Δ*bcstc3* construct	This study
***Bcstc3*-3R**	GCGGATAACAATTTCACACAGGAAACAGCAT CTGGCGCAATAAGGAGTG	pRS426-3R – *bcstc3*-3'	Cloning – amplification of 3' flank for Δ*bcstc3* construct	This study
***Bcstc3*-3F**	CTCCTTCAATATCATCTTCTGTCTCCGACCTAC TGAAATAGCTTCCTTGCAC	P*trpC* – *bcstc3*-3'	Cloning – amplification of 3' flank for Δ*bcstc3* construct	This study
***Bcstc3*-hi5F**	GGAGGACGCATATAGATCCA	*Bcstc3*-5'	Diagnostic PCR – homologous integration at *bcstc3*-5'	This study
***Bcstc3*-hi3R**	GTGGAGGAATCTGGACTTGA	*Bcstc3*-3'	Diagnostic PCR – homologous integration at *bcstc3*-3'	This study
***Bcstc3*-WT-F**	CTTGCCAAACGACAGCTCCG	*Bcstc3* ORF	Diagnostic PCR – detection of *bcstc3* alleles	This study
***Bcstc3*-WT-R**	CCCGTACAGATGAAAATCCC	*Bcstc3* ORF	Diagnostic PCR – detection of *bcstc3* alleles	This study
**PactA-amp-F**	GGGGTTGATAAATTAAGACGTTAAGTCCTAT TACCGTATTCATTTGG	PactA – bcstc3-5'	Cloning – amplification of PactA for overexpression construct and Diagnostic PCR – homologous integration at bcniaD-3'	This study
**PactA-PtrpC-R**	GCCCAAAAAATGCTCCTTCAATGTCACTAGTT GTGCGTCCTCTTCTGCCTACCCA	PtrpC – PactA	Cloning – amplification of PactA for overexpression construct	[1]
**TtrpC-Bcstc3-F**	TGACATGGAGCTATTAAATCACTAAGCGGCC GCTGGTACTTCTTTAAGCTTAGT	TtrpC – bcstc3	Cloning – amplification of bcstc3 locus for overexpression construct	This study
**PactA-*Bcstc3*-R**	GACTTAACGTCTTAATTTATCAACCCCGAACT TTCCAAATGAATACGGTAATAG	PactA – bcstc3	Cloning – amplification of bcstc3 locus for overexpression construct and Diagnostic PCR – homologous integration at bcniaD-5'	This study
***BcniaD*-5F**	GTGAATGGGATTCATTGTTTATTTC	*BcniaD-5'*	Cloning – amplification of overexpression fragment	[1]
***BcniaD*-3R**	GCATTGGATTAATAATTGTTGCTAAGC	*BcniaD*-3'	Cloning – amplification of overexpression fragment	[1]
***BcniaD*-hi5F**	CGCATATCAGCATATCGAGATGTCC	*BcniaD* locus	Diagnostic PCR – homologous integration at *bcniaD*-5'	[1]
***BcniaD*-hi3R**	GAGTACCCATCCGATGGAGTTGTTG	*BcniaD* locus	Diagnostic PCR – homologous integration at *bcniaD*-3'	[1]
BcniaD-WT-F	GCCACAGACTCCGCCAGATTCTAATG	BcniaD locus	Diagnostic PCR – detection of BcniaD alleles	[1]
BcniaD-WT-R	CAACCATTTCACGCTGCGACCACC	BcniaD locus	Diagnostic PCR – detection of BcniaD alleles	[1]
**Bcstc1fw**	TATGAGCAGCAACAGTACGG	Bcstc1-5'	mRNA level quantification of the gene *Bcstc1*	[2,3]
**Bcstc1rv**	GCTGCTTCAATTCCTGGGTG	Bcstc1-3'	mRNA level quantification of the gene *Bcstc1*	[2,3]
**Bcstc2fw**	CATCATCGATGATTGCGTCC	Bcstc2-5'	mRNA level quantification of the gene *Bcstc2*	[2,3]
**Bcstc2rv**	GAACCTTCATCCAACCATCC	Bcstc2-3'	mRNA level quantification of the gene *Bcstc2*	[2,3]
**Bcstc3fw**	GGAAAGCAAATGAGAGGGCG	Bcstc3-5'	mRNA level quantification of the gene *Bcstc3*	[2,3]
**Bcstc3rv**	TGTCCATTGGTTAGCATGGC	Bcstc3-5'	mRNA level quantification of the gene *Bcstc3*	[2,3]
**Bcstc4fw**	ATCCAGTACCAGAGGTACCC	Bcstc4-5'	mRNA level quantification of the gene *Bcstc4*	[2,3]
**Bcstc4rv**	CACGCGTACGCTATTTACGC	Bcstc4-3'	mRNA level quantification of the gene *Bcstc4*	[2,3]
**Bcstc5fw**	GCACGAGTTGTTAAGAAGG	Bcstc5-5'	mRNA level quantification of the gene *Bcstc5*	[2,3]
**Bcstc5rv**	CCATCTGAATTGCCTTCTGC	Bcstc5-3'	mRNA level quantification of the gene *Bcstc5*	[2,3]
**Bcstc7fw**	TGTATGAGGTGCTGCACTGG	Bcstc7-5'	mRNA level quantification of the gene *Bcstc7*	[2,3]
**Bcstc7rv**	GACTTCATTCTGATGACGCG	Bcstc7-3'	mRNA level quantification of the gene *Bcstc7*	[2,3]
**BcactAfw**	TTTGAGACCTTCAACGCCCC	BcactA-5'	mRNA level quantification of the gene *BcactA*	[2,3]
**BcactArv**	ACGTGAGTAACTCCGTCACC	BcactA-3'	mRNA level quantification of the gene *BcactA*	[2,3]
**Bctubfw**	TCCTTTCGGTCAACTCTTCCG	Bctub-5'	mRNA level quantification of the gene *Bctub*	[2,3]
**Bctubrv**	CACCCTCAGTGTTAATGACCC	Bcstub-3'	mRNA level quantification of the gene *Bctub*	[2,3]

References

1. Schumacher, J. Tools for *Botrytis cinerea*: New expression vectors make the gray mold fungus more accessible to cell biology approaches. *Fungal Genet. Biol.* **2012**, *49*, 483–497, doi:10.1016/j.fgb.2012.03.005.
2. Pinedo, C.; Moraga, J.; Barua, J.; González-Rodríguez, V.E.; Aleu, J.; Durán-Patrón, R.; Macías-Sánchez, A.J.; Hanson, J.R.; Viaud, M.; Hernández-Galán, R.; et al. Chemically induced cryptic sesquiterpenoids and expression of sesquiterpene cyclases in *Botrytis cinerea* revealed new sporogenic (+)-4- Epieremophil-9-en-11-ols. *ACS Chem. Biol.* **2016**, *11*, 1391–1400, doi:10.1021/acschembio.5b00931.
3. Izquierdo-Bueno, I.; González-Rodríguez, V.E.; Simon, A.; Dalmais, B.; Pradier, J.; Le Pêcheur, P.; Mercier, A.; Walker, A.; Garrido, C.; Collado, I.G.; et al. Biosynthesis of abscisic acid in fungi: identification of a sesquiterpene cyclase as the key enzyme in *Botrytis cinerea*. *Environ. Microbiol.* **2018**, *20*, 2469–2482, doi:10.1111/1462-2920.14258.
